# Fermentative production of Vitamin E tocotrienols in *Saccharomyces cerevisiae* under cold-shock-triggered temperature control

**DOI:** 10.1038/s41467-020-18958-9

**Published:** 2020-10-14

**Authors:** Bin Shen, Pingping Zhou, Xue Jiao, Zhen Yao, Lidan Ye, Hongwei Yu

**Affiliations:** 1 Key Laboratory of Biomass Chemical Engineering (Education Ministry), College of Chemical and Biological Engineering, Zhejiang University, 310027 Hangzhou, China; 2 Institute of Bioengineering, College of Chemical and Biological Engineering, Zhejiang University, 310027 Hangzhou, China; 3 College of Bioscience and Biotechnology, Yangzhou University, 225009 Yangzhou, China

**Keywords:** Metabolic engineering, Applied microbiology, Synthetic biology

## Abstract

The diverse physiological functions of tocotrienols have listed them as valuable supplementations to α-tocopherol-dominated Vitamin E products. To make tocotrienols more readily available, tocotrienols-producing *S. cerevisiae* has been constructed by combining the heterologous genes from photosynthetic organisms with the endogenous shikimate pathway and mevalonate pathway. After identification and elimination of metabolic bottlenecks and enhancement of precursors supply, the engineered yeast can produce tocotrienols at yield of up to 7.6 mg/g dry cell weight (DCW). In particular, proper truncation of the N-terminal transit peptide from the plant-sourced enzymes is crucial. To further solve the conflict between cell growth and tocotrienols accumulation so as to enable high-density fermentation, a cold-shock-triggered temperature control system is designed for efficient control of two-stage fermentation, leading to production of 320 mg/L tocotrienols. The success in high-density fermentation of tocotrienols by engineered yeast sheds light on the potential of fermentative production of vitamin E tocochromanols.

## Abstract (web summary)

Tocotrienols are valuable supplementations to α-tocopherol-dominated Vitamin E products. Here, the authors engineer baker’s yeast by combining the heterologous genes from photosynthetic organisms with the endogenous pathway for the production of tocotrienols under cold-shock-triggered temperature control.

## Introduction

Vitamin E, an essential nutrient in the human diet, is a generic term that refers to α, β, γ, δ-tocopherols and tocotrienols, all of which are amphipathic molecules with a hydrophobic isoprenoid-derived hydrocarbon tail and a polar aromatic head obtained from the shikimate pathway^1^. The major structural difference of tocotrienols from tocopherols lies in the unsaturated isoprenoid side chain containing three double bonds, which would potentially enhance their deeper penetration into tissues that have saturated fatty layers such as the brain and liver^2^. Similar to α-tocopherol, the most extensively studied vitamin E form, tocotrienols are excellent antioxidants^3,4^. Moreover, studies have shown some unique functions associated to tocotrienols that are often not exhibited by tocopherols, such as lowering cholesterol by inhibiting the activity of 3-hydroxy-3-methylglutaryl-coenzyme A (HMG-CoA) reductase^5^, neuroprotective function by suppressing early activation of c-Src kinase and 12-lipoxygenase^6^ and potent radioprotectant against radiation damage^7^. These properties make tocotrienols valuable supplementations to the current α-tocopherol-based products.

Although racemic α-tocopherol has been chemically synthesized since 1938^8^, commercial synthesis of tocotrienols has not been achieved yet, ascribed to the complicated steps, low yields and purity^9^. In nature, vitamin E is exclusively synthesized in oxygenic photosynthetic organisms^10,11^, and tocotrienols are relatively rarer distributed in nature sources and generally found in the seeds of monocotyledons and fruits of some dicotyledons^12^. Currently, natural tocotrienols are mainly derived from crude palm oil which contains up to 800 mg/kg of tocotrienols^13^. Recently, metabolic engineering efforts in soybean seeds have turned soybean into a viable platform for producing high levels of vitamin E tocochromanols^14,15^. Meanwhile, recent advances in synthetic biology have enabled heterologous biosynthesis of various natural products in model microorganisms^16–19^. The synthetic pathway of vitamin E has been determined by mutational analysis and in vitro enzyme characterization^20^, which lays basis for its heterologous biosynthesis. The first effort on microbial biosynthesis of vitamin E tocochromanols produced 15 μg/g DCW of δ-tocotrienol in engineered *Escherichia coli*^21^. However, the low yield and biosafety concerns with *E. coli* discourage its further application. Considering the GRAS (generally regarded as safe) status of *Saccharomyces cerevisiae* and its outstanding performance in heterologous production of terpenoids and aromatic compounds^22,23^, construction of efficient yeast cell factory may be a viable approach to sustainable tocotrienols production. De novo biosynthesis of δ-tocotrienol in *S. cerevisiae* with a titer of 4.1 mg/L has recently been reported^24^.

Tocotrienols-producing yeast strains could be constructed by assembly of the synthetic pathway genes sourced from photosynthetic organisms in *S. cerevisiae*. However, when plant-derived proteins are expressed in microorganisms, their expression level and enzyme activity are often limited due to improper folding and presence of transit peptides, leading to metabolic bottlenecks in the pathway^25,26^. Moreover, the compatibility of the heterologous pathway with the native metabolism should also be taken into consideration, such as concerns of cofactor imbalance, precursor competition, redox imbalance, and intermediate toxicity, all of which would result in low biosynthetic efficiency^27–29^. Therefore, to construct an unimpeded tocotrienols synthetic pathway in *S. cerevisiae*, efforts are needed to debug the target pathway and balance it with the related native pathways.

In order to achieve high production of tocotrienols, the conflict between product accumulation and cell growth which is commonly encountered in biosynthesis^30^ should also be addressed. In previous efforts towards high production of carotenoids, we have developed glucose-responsive and temperature-responsive regulation systems to separate the production stage from the growth stage^31,32^. In particular, the temperature-responsive control system based on Gal4 engineering could lead to tight regulation of two-stage fermentation^32,33^. However, trade-off between activity and temperature sensitivity occurred during directed evolution of Gal4, and the lower activity of the temperature-sensitive mutant Gal4M9 would limit the production of target metabolite. Moreover, the fermentation temperature should be maintained at the permissive temperature of Gal4M9 (24 °C) during the whole product accumulation stage, raising the issue of high energy consumption. To achieve high-density fermentation of tocotrienols in a more energy-saving manner, redesign of the dynamic control system is required.

In this study, we set out to construct tocotrienols-producing yeast by gene cloning from various photosynthetic organisms, followed by codon optimization and protein truncation to facilitate their functional expression in *S. cerevisiae*. Subsequently, individual and combined optimization of the heterologous pathway and the two precursor pathways is performed for bottleneck elimination and balance reconstruction. Finally, to enable high-density fermentation of tocotrienols, a cold-shock-triggered temperature control system is designed by placing the wild-type Gal4 under control of Gal4M9 to simultaneously regulate the expression and activity of the transcriptional activator upon temperature shift and to ensure continuous supply of Gal4 after changing the temperature back to 30 °C. In this way, the activity reduction of Gal4M9 can be compensated and the P_*GAL*_-driven biosynthetic pathway could be kept switched on without the need of sustaining a low fermentation temperature.

## Results

### Construction of tocotrienols biosynthetic pathway in yeast

According to the vitamin E synthetic pathway in plants, tocotrienols are formed from two precursors, homogentisic acid (HGA) synthesized from 4-hydroxyphenylpyruvate (4-HPP) under catalysis of 4-hydroxyphenylpyruvate dioxygenase (HPPD)^34^ and geranylgeranyl pyrophosphate (GGPP) derived from 2C-Methyl-D-erythritol-4-phosphate (MEP) pathway. GGPP is transferred to HGA by homogentisate geranylgeranyl transferase (HGGT) to synthesize 2-methyl-6-geranylgeranyl benzoquinol (MGGBQ), which is subsequently transformed into tocotrienols via methylation and/or cyclization. MPBQ methyltransferase (MPBQMT) is in charge of catalyzing MGGBQ methylation to produce 2,3-dimethyl-5-geranylgeranyl-benzoquinone (DMGGBQ), the second ring of which could be closed by tocopherol cyclase (TC) to synthesize γ-tocotrienol. TC can also catalyze the ring closing reaction on MGGBQ to generate δ-tocotrienol. γ-TMT further methylates δ-tocotrienol and γ-tocotrienol to produce β-tocotrienol and α-tocotrienol, respectively^35^.

**Fig. 1:**
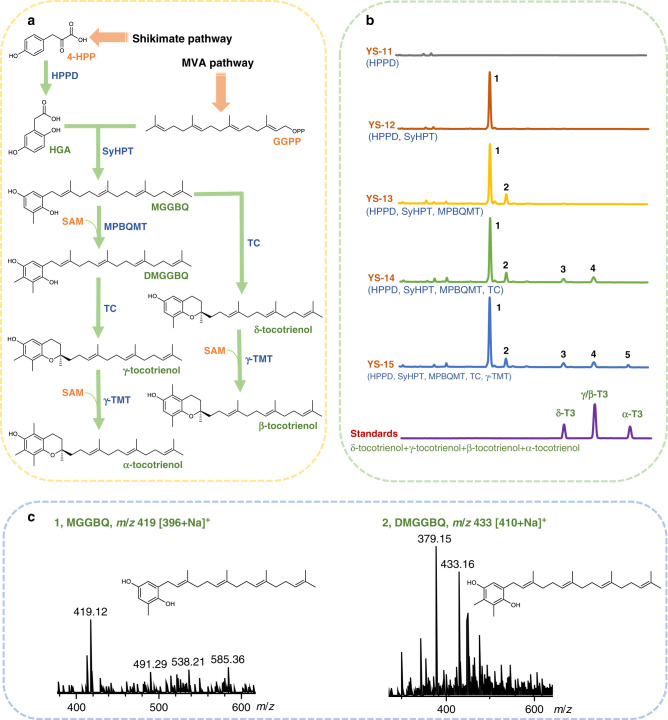
Construction of tocotrienols biosynthetic pathway in *S. cerevisiae*. **a** Proposed tocotrienols synthetic pathway in *S. cerevisiae*. MVA pathway mevalonate pathway, 4-HPP 4-hydroxyphenylpyruvate; HGA homogentisic acid, GGPP geranylgeranyl pyrophosphate, MGGBQ 2-methyl-6-geranylgeranyl benzoquinol, DMGGBQ 2,3-dimethyl-5-geranylgeranyl-benzoquinone, HPPD 4-hydroxyphenylpyruvate dioxygenase, SyHPT homogentisate phytyltransferase, MPBQMT 2-methyl-6-phytylbenzoquinol methyltransferase, TC tocopherol cyclase, γ-TMT γ-tocopherol methyltransferase. **b** HPLC spectra of *S. cerevisiae* strains expressing the heterologous pathway genes. The retention time of γ-tocotrienol and β-tocotrienol was the same under the chromatographic conditions used. T3, tocotrienol. 1. MGGBQ; 2. DMGGBQ; 3. δ-T3; 4. γ/β-T3; 5. α-T3. **c** LC-MS analysis of MGGBQ and DMGGBQ in positive ion mode. Source data are provided as a Source Data file.

In order to construct a complete biosynthetic pathway of tocotrienols in *S. cerevisiae*, the endogenous mevalonate (MVA) pathway leading to formation of GGPP and shikimate pathway generating 4-HPP should be extended by five exogenous enzymes from photosynthetic organisms, namely HPPD, HGGT, MPBQMT, TC and γ-TMT (Fig. 1a). These enzymes were first cloned from *Arabidopsis thaliana* and expressed in *S. cerevisiae* using EGFP as a reporter. Fluorescence was detected for HPPD, MPBQMT, γ-TMT and codon-optimized TC, indicating successful expression. However, even after codon optimization, no fluorescence signal could be detected for HGGT. In some photosynthetic organisms, HGGT and homogentisate phytyltransferase (HPT) catalyzing the formation of tocopherol precursor are isoenzymes^11^. Further efforts in heterologous expression of HPT/HGGT from 12 plants (Supplementary Table 1) failed, either with or without codon optimization, so we turned to algae. The enzymes reported with HPT/HGGT activity in the eukaryotic alga *Chlamydomonas reinhardtii* and the cyanobacteria *Synechocystis* were cloned, and only the codon-optimized HPT from *Synechocystis* sp. PCC6803 (SyHPT) was successfully expressed. In this way, all heterologous enzymes required for assembly of tocotrienols synthetic pathway in yeast were obtained.

Functional expression of these enzymes was verified by detection of the respective metabolic intermediates (Fig. 1b). When *HPPD*, directly cloned or codon-optimized, was integrated into the genome of YS-01 (BY4741, Δ*GAL80, HMG1*::*tHMG1*-P_*GAL10*_-P_*GAL1*_-*CrtE*) with modified *GAL* regulation system^31^ and strengthened MVA pathway^36,37^, 10.2 mg/L and 11.1 mg/L of HGA was detected respectively after 72 h incubation, demonstrating functional expression of the *A. thaliana* HPPD in *S. cerevisiae*. After addition of 0.1% (w/v) tyrosine into the medium, HGA titers were increased to 30.1 mg/L and 37.3 mg/L, respectively. When the codon-optimized *SyHPT* was integrated into the genome of YS-11 (YS-01, Δ*HO*::P_*GAL1*_-*HPPD*), a peak with masses of 419[M + Na]^+^ and 395[M-H]^−^ emerged (Fig. 1c), which was in consistence with those of MGGBQ synthesized in the engineered *E. coli* expressing heterologous *crtE*, *hpd* and *hpt* genes^38^, confirming the functional expression of SyHPT in YS-12 (YS-11, Δ*DPP1*::P_*GAL1*_-*SyHPT*). In comparison, when the *HPT/HGGT* genes from other photosynthetic organisms were introduced into YS-11 either via genomic integration or high-copy-number episomal plasmid (pESC-URA), no MGGBQ was detected. When the synthetic pathway was further extended by integration of the codon-optimized *MPBQMT* into the genome of YS-12, a new peak with masses of 433[M + Na]^+^ and 409[M-H]^−^ corresponding to DMGGBQ was detected in the resulting strain YS-13 (YS-11, Δ*DPP1*::*MPBQMT*-P_*GAL10*_-P_*GAL1*_-*SyHPT*) (Fig. 1c). Subsequent introduction of the codon-optimized TC led to formation of δ-tocotrienol and γ-tocotrienol in the resulting strain YS-14 (YS-13, Δ*GAL1-7*::P_*GAL1*_-*TC*), as identified by LC-MS with reference to the commercial standards (Supplementary Fig. 1). Extension of the pathway by introducing the second methyltransferase, the codon-optimized γ-TMT, successfully delivered α-tocotrienol in YS-15 (YS-13, Δ*GAL1-7*::*γ-TMT*-P_*GAL10*_-P_*GAL1*_-*TC*). After culturing in YPD medium (with addition of 0.1% Tyr) for 96 h, the total tocotrienols yield of YS-15 reached 313 μg/g DCW, including 67.6 μg/g DCW of δ-tocotrienol, 172.9 μg/g DCW of γ/β-tocotrienol and 72.3 μg/g DCW of α-tocotrienol.

### Identification and elimination of rate-limiting steps

**Fig. 2:**
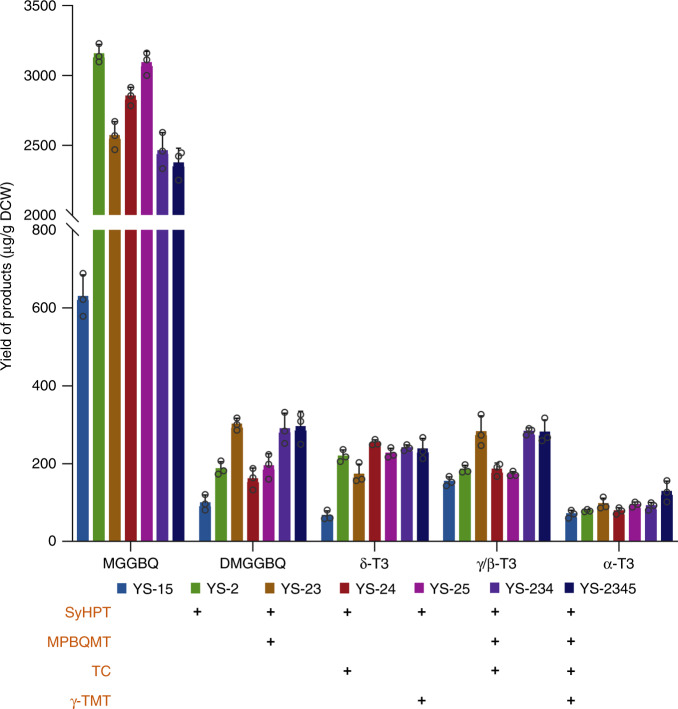
Product profiles of the yeast strains overexpressing different enzyme combinations of the tocotrienols synthetic pathway. Columns of different colors represent different strains, and the horizontal axis shows different metabolites in the tocotrienols synthetic pathway. The enzymes overexpressed with an extra gene copy in each strain are indicated with ‘+’. All values presented are the means of three biological replicates, and error bars represent standard deviations. Source data are provided as a Source Data file.

Based on strain YS-15, rate-limiting steps were identified by overexpressing the five exogenous enzymes one by one. Upon overexpression of SyHPT, significant accumulation of MGGBQ was detected in strain YS-2 (YS-15, *GAL80*::P_*GAL1*_-*SyHPT*), which was approximately 4-fold higher than that of YS-15. However, overexpression of the other four enzymes in YS-15 did not lead to obvious changes in the corresponding metabolites, which may be resulted from the limited supply of MGGBQ when only one copy of *SyHPT* was expressed. In the background of SyHPT overexpression, MPBQMT, TC and γ-TMT were overexpressed separately and in combinations, generating strains YS-23 (YS-15, Δ*GAL80*::*MPBQMT*-P_*GAL10*_-P_*GAL1*_-*SyHPT*), YS-24 (YS-15, Δ*GAL80*::*TC*-P_*GAL10*_-P_*GAL1*_-*SyHPT*), YS-25 (YS-15, Δ*GAL80*::*γ-TMT*-P_*GAL10*_-P_*GAL1*_-*SyHPT*), YS-234 (YS-23, Δ*Ty4*::P_*GAL1*_-*TC*) and YS-2345 (YS-23, Δ*Ty4*::*γ-TMT*-P_*GAL10*_-P_*GAL1*_-*TC*) (Fig. 2). Compared with YS-2, DMGGBQ accumulation in YS-23 was increased by 83%, but the γ-tocotrienol production in YS-24 was not obviously improved whereas the δ-tocotrienol yield was improved by 28% instead. The similar product profiles of YS-23 and YS-234 suggested the more important role of the methyltransferase MPBQMT in flux control as compared to the cyclase, whereas the obvious accumulation of MGGBQ indicated sole overexpression of MPBQMT was insufficient to eliminate the bottleneck. Even when the two methyltransferases and the cyclase were overexpressed together, production of tocotrienols in YS-2345 was still not significantly improved with MGGBQ remaining as the major metabolite, raising the concern that the biosynthetic efficiency might have been limited by the low catalytic activity of the *A. thaliana* enzymes. Therefore, these enzymes were also cloned from the fruit of *E. guineensis* which is rich in tocotrienols and the cyanobacteria *Synechocystis* sp. PCC6803, respectively, and used for pathway construction in *S. cerevisiae*. However, all of them showed lower activity than those of *A. thaliana*, even after codon optimization (Supplementary Fig. 2).

**Fig. 3:**
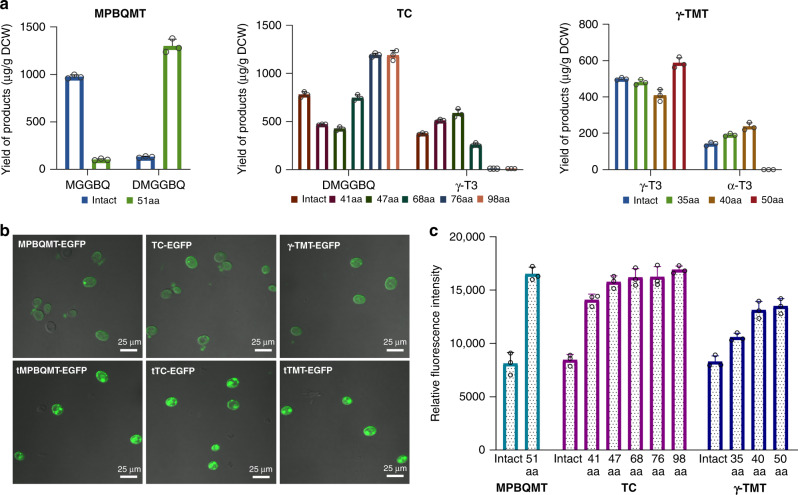
Prediction and excision of chloroplast transit peptide in MPBQMT, TC, and γ-TMT. **a** Effect of excising predicted transit peptides with different lengths on the corresponding metabolites of MPBQMT, TC, and γ-TMT. **b** Laser confocal microscopy of strains expressing fusion proteins of EGFP and MPBQMT, TC, and γ-TMT before and after removal of the transit peptide. Names without prefix refer to the full-length proteins, whereas names prefixed with ‘t’ refer to the truncated ones. The tests were repeated three times independently with similar results. **c** Changes in relative fluorescence intensity divided by OD_600_ of strains expressing EGFP fused with MPBQMT, TC, and γ-TMT before and after truncation of different length of the N-terminal sequence. All values presented are the means of three biological replicates, and error bars represent standard deviations. Source data are provided as a Source Data file.

Considering that MPBQMT, TC and γ-TMT are all chloroplast-localized in plants^39,40^, the presence of transit peptides may prevent them from proper expression in *S. cerevisiae*. Therefore, we predicted the transit peptides for each enzyme and created truncated proteins based on the predicted slicing site with high CS score. For MPBQMT, a chloroplast transit peptide with a length of 51 aa was predicted. When this N-terminal sequence was excised, the accumulation of DMGGBQ was increased by about 9 times. For TC and γ-TMT, since the truncation of the predicted transit peptides (98 aa and 50 aa respectively) led to activity loss, four and two additional N-terminal sequences with relatively high CS scores and different lengths were tested respectively. Excision of the 47 aa and 40 aa N-terminal sequences of TC and γ-TMT led to the best results, with 56 and 67% improvement in the accumulation of corresponding metabolites, respectively (Fig. 3a). To check whether the activity improvement of these enzymes was resulted from enhanced protein expression or altered cellular localization, the influence of transit peptide excision was analyzed using EGFP as a reporter. No change in localization was observed under laser confocal microscopy (Fig. 3b), whereas the fluorescence intensity was obviously increased after removal of the transit peptides. The relative fluorescence intensity increased with the increase of the truncated sequence length before reaching a peak value, and there was about 103, 86, and 58% increase in the expression levels of the truncated MPBQMT, TC and γ-TMT with highest activities, respectively (Fig. 3c).

**Fig. 4:**
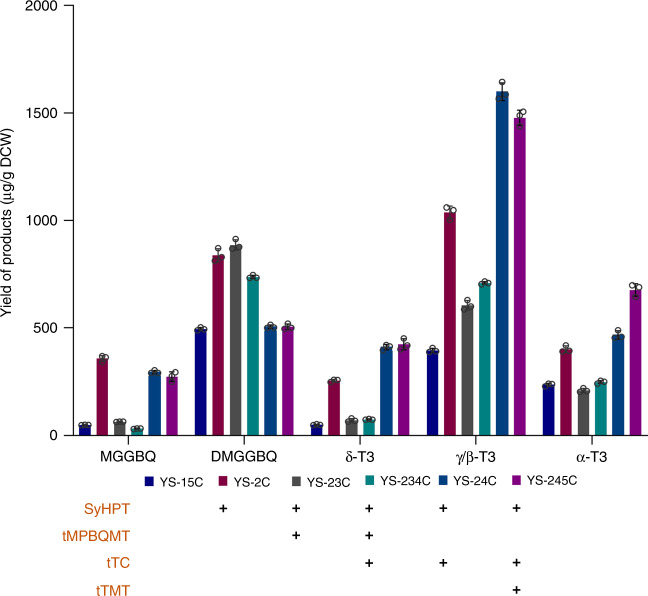
Product profiles of strains overexpressing the truncated rate-limiting enzymes. Columns of different colors represent different strains, and the horizontal axis shows different metabolites in the tocotrienols synthetic pathway. The enzymes overexpressed with an extra gene copy in each strain are indicated with ‘+’. All values presented are the means of three biological replicates, and error bars represent standard deviations. Source data are provided as a Source Data file.

By replacing the full-length MPBQMT, TC and γ-TMT with the truncated enzymes (namely tMPBQMT, tTC and tTMT), we constructed strain YS-15C (YS-11, Δ*DPP1*::*tMPBQMT*-P_*GAL10*_-P_*GAL1*_-*SyHPT*, Δ*GAL1-7*::*tTMT*-P_*GAL10*_-P_*GAL1*_-*tTC*) with total tocotrienols yield of 680.4 μg/g DCW, which was 2.2 fold as much as that of the control strain YS-15. To further improve tocotrienols production, we overexpressed the rate-limiting enzymes in YS-15C, generating YS-2C (YS-15C, Δ*GAL80*::P_*GAL1*_-*SyHPT*), YS-23C (YS-15C, Δ*GAL80*::*tMPBQMT*-P_*GAL10*_-P_*GAL1*_-*SyHPT*), YS-24C (YS-15C, Δ*GAL80*::*tTC*-P_*GAL10*_-P_*GAL1*_-*SyHPT*), YS-245C (YS-2C, Δ*Ty4*::*tTMT*-P_*GAL10*_-P_*GAL1*_-*tTC*) and YS-234C (YS-23C, Δ*Ty4*::P_*GAL1*_-*tTC*) (Fig. 4). Compared with YS-15C, the tocotrienols production of YS-2C was further improved by 1.5 fold, and the MGGBQ accumulation was increased by about 5 times. Overexpression of tMPBQMT was expected to accelerate the conversion of MGGBQ, however it led to decrease in both biomass and tocotrienols yield of YS-23C as compared to YS-2C. Overexpression of tTMT in YS-24 led to highest yield of α-tocotrienol, which was 96% higher than that of YS-2C. The total tocotrienols yield of YS-245C was approximately 8 times higher than that of the initial strain YS-15, reaching 2.6 mg/g DCW, including 423.5, 1477.9 and 677.4 μg/g DCW of δ-, γ/β- and α-tocotrienol, respectively.

### Enhancement of precursor supply

**Fig. 5:**
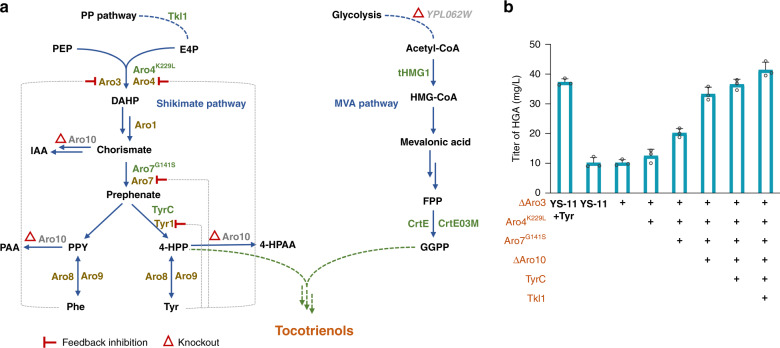
Enhancement of precursor supply for tocotrienols production. **a** Strategy for modification of the shikimate pathway and MVA pathway to enhance precursor supply. The genes that are overexpressed are highlighted in green, and those knocked out are shown in gray. PP Pathway pentose phosphate pathway, PEP phosphoenolpyruvate, E4P D-erythrose-4-phosphate, DAHP 7P-2-dehydro-3-deoxy-D-arabino-heptonate, 4-HPP 4-hydroxy-phenylpyruvate, PPY phenylpyruvate, Tyr tyrosine, Phe phenylalanine, MVA pathway mevalonic acid pathway, HMG-CoA 3-hydroxy-3-methyl-glutaryl-CoA, FPP farnesyl pyrophosphate, GGPP geranylgeranyl diphosphate, IAA indole acetaldehyde, PAA phenylacetaldehyde, 4-HPAA 4-hydroxyphenylacetaldehyde, Tkl1 transketolase, Aro4 3-deoxy-D-arabino-heptulosonate-7-phosphate synthase, Aro7 chorismate mutase, Tyr1/TyrC cyclohexadienyl dehydrogenase, Aro10 phenylpyruvate decarboxylase, Aro8/Aro9 tyrosine aminotransferase, tHMG1 truncated 3-hydroxy-3-methyl-glutaryl reductase, CrtE geranylgeranyl diphosphate synthase. **b** Effect of stepwise optimization of shikimate pathway on HGA accumulation in YS-11. YS-11+Tyr indicates YS-11 cultured with exogenously addition of tyrosine. All values presented are the means of three biological replicates, and error bars represent standard deviations. Source data are provided as a Source Data file.

**Fig. 6:**
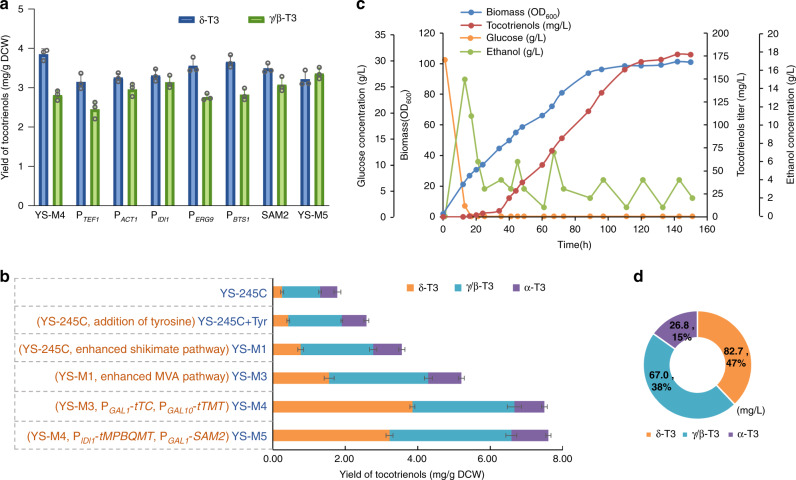
Comprehensive pathway optimization and fed-batch fermentation. **a** Effect of tMPBQMT overexpression under different promoters and SAM2 overexpression on δ-tocotrienol and γ-tocotrienol yields. In YS-M4, tMPBQMT was expressed under P_*GAL10*_. In YS-M5, tMPBQMT was overexpressed under P_*IDI1*_ and SAM2 was overexpressed under P_*GAL1*_. **b** Yield and composition of tocotrienols in the engineered yeast strains after stepwise pathway optimization. **c** Time courses of cell growth, tocotrienols production and consumption of carbon sources during fed-batch fermentation of tocotrienols by YS-M5(+). **d** Composition of tocotrienols produced by the end of YS-M5(+) fermentation. T3, tocotrienol. All values presented are the means of three biological replicates, and error bars represent standard deviations. Source data are provided as a Source Data file.

Sufficient supply of precursors is essential for efficient biosynthesis. Improvement of HGA production by addition of exogenous tyrosine which can reversibly generate 4-HPP by tyrosine aminotransferase Aro8/Aro9^41^ implied shortage of precursor supply from the shikimate pathway in the engineered yeast (Fig. 5b). The endogenous shikimate pathway in YS-245C was therefore strengthened to get rid of the dependence on exogenous tyrosine (Fig. 5a). First, the effect of shikimate pathway optimization was investigated in YS-11, where HPPD was introduced to produce HGA. The tyrosine-insensitive mutants of 3-deoxy-D-arabino-heptulosonate-7-phosphate synthase (Aro4^K229L^) and chorismate mutase (Aro7^G141S^)^42^ were introduced together with the codon-optimized tyrosine-insensitive cyclohexadienyl dehydrogenase (TyrC) from *Zymomonas mobilis*^43^ to relieve feedback inhibition of tyrosine, and the endogenous transketolase (Tkl1) was overexpressed to increase the pool of the precursor erythrose-4-phosphate (E4P)^44^. Meanwhile, phenylpyruvate decarboxylase (Aro10) was knocked out to block the aromatic amino acid degradation pathway^45^. As a result, the HGA titer in YS-11 was increased by 2.9 times (Fig. 5b). The optimized shikimate pathway was then introduced into the tocotrienols-producing yeast YS-245C. The resulting strain YS-M1 (YS-245C, Δ*ARO3*::*A*ro*7*^*G141S*^-P_*GAL10*_-P_*GAL1*_-*Aro4*^*K229L*^, Δ*ARO10*::*TyrC*-P_*GAL10*_-P_*GAL1*_-*TKL1*) produced 3.6 mg/g DCW of tocotrienols in the absence of exogenous tyrosine, which was 1.4 times as much as that of YS-245C with exogenously added tyrosine (Fig. 6b).

As for the supply of the other key precursor GGPP from the MVA pathway, the well-known rate-limiting steps had been strengthened by overexpressing tHMG1 and introducing the CrtE from *Xanthophyllomyces dendrorhous* in all the above strains. To further enhance GGPP supply so as to reconstruct the flux balance after respective enhancement of the shikimate pathway and the heterologous tocotrienols synthetic pathway in YS-M1, *YPL062W* was deleted to increase the cytosolic acetyl-CoA pool^46^, and the positive mutant of CrtE (CrtE03M) previously obtained by directed evolution^47^ was introduced (Fig. 5a), constructing YS-M3 (YS-M1, Δ*YPL062W*::P_*GAL10*_-*CrtE03M*). As compared with YS-245C, the total tocotrienols production of YS-M3 was doubled after engineering of the precursor pathways, reaching 5.20 mg/g DCW, including 1.6, 2.7 and 0.8 mg/g DCW of δ-, γ/β- and α-tocotrienol, respectively (Fig. 6b).

### Comprehensive pathway optimization

After strengthening the precursor pathways, the accumulation of intermediates in the tocotrienols synthetic pathway was significantly elevated. Considering the relatively high accumulation of MGGBQ and DMGGBQ in YS-M3, tTC and tTMT were further overexpressed to pull the metabolic flux towards tocotrienols formation. In the resulting strain YS-M4 (YS-M3, Δ*LPP1*::*tTMT*-P_*GAL10*_-P_*GAL1*_-*tTC*), its precursors MGGBQ and DMGGBQ were almost depleted (Supplementary Fig. 3), δ-tocotrienol increased markedly to 3.9 mg/g DCW, and the total tocotrienols increased to 7.5 mg/g DCW (Fig. 6b).

To improve the synthesis of the methylated tocotrienol isomers, the catalytic capacities of the methyltransferases need to be enhanced. Considering that overexpression of tMPBQMT under P_*GAL10*_ in YS-2C resulted in decreased biomass implying excessive metabolic burden (Fig. 4), we tested five constitutive promoters with different strengths, P_*TEF1*_, P_*ACT1*_, P_*IDI1*_, P_*ERG9*_, and P_*BTS1*_, to find a promoter with suitable strength to overexpress tMPBQMT. The medium-strength P_*IDI1*_ was found to be the most suitable promoter, with 12% higher γ/β-tocotrienol and 15% lower δ-tocotrienol yield as compared to YS-M4 (Fig. 6a), and without affecting the biomass. The efficiency of methyl transfer is not only determined by the activity of methyltransferase, but also depends on sufficient supply of S-adenosyl-L-methionine (SAM) as the methyl donor^48,49^. Overexpression of the SAM synthetase SAM2 has been reported to greatly enhance SAM accumulation in *S. cerevisiae*^50,51^. When we overexpressed SAM2 in YS-M4, the yields of γ/β-tocotrienol and α-tocotrienol increased by 10 and 20%, respectively (Fig. 6a). By overexpressing *tMPBQMT* under P_*IDI*_ and *SAM2* under P_*GAL1*_ in YS-M4, the resulting strain YS-M5 (YS-M4, Δ*YPL062W*:: P_*GAL1*_-*SAM2*, Δ*Ty4*::P_*IDI1*_-tMPBQMT) produced 7.60 mg/g DCW of the total tocotrienols, including 3.2, 3.4 and 1.0 mg/g DCW of δ-, γ/β- and α-tocotrienol, respectively (Fig. 6b).

After complementation of auxotrophic markers in YS-M5 to generate the prototrophic haploid strain YS-M5(+), fed-batch fermentation was performed in a 5L bioreactor with glucose feeding in cell growth stage and ethanol feeding in product accumulation stage. However, the strain grew slowly, and the logarithmic growth period was short, implying high metabolic stress. Finally, the maximum biomass was merely 100 OD_600_, and the tocotrienols titer was 176.7 mg/L (Fig. 6c, d).

### Design of a cold-shock-triggered temperature control system

**Fig. 7:**
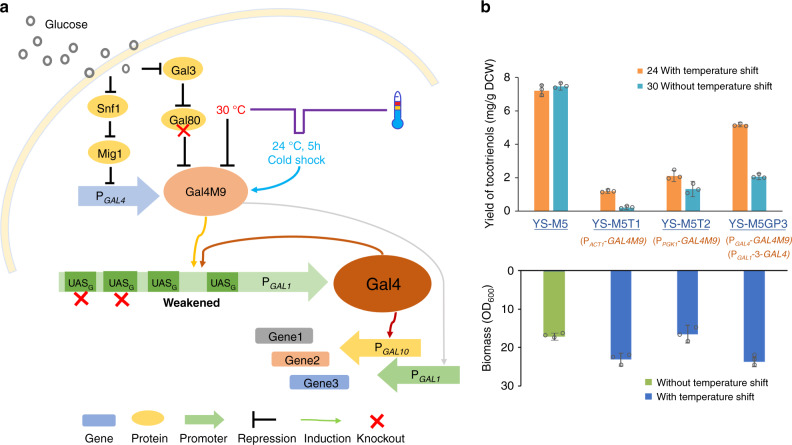
Design of the cold-shock-triggered temperature control system in *S. cerevisiae*. **a** Design of the cold-shock-triggered temperature control system based on Gal4M9 and wild-type Gal4. After deletion of *GAL80*, the P_*GAL4*_-driven Gal4M9 is expressed upon glucose depletion, and its activity is controlled by temperature. When the temperature is changed from 30 to 24 °C, the activated Gal4M9 switches on the expression of the wild-type Gal4 under control of the weakened P_*GAL1*_-3, and the accumulated Gal4 activates the expression of P_*GAL*_-driven genes including its own encoding gene. Even when the temperature is switched back to 30 °C after cold shock, the pathway can be kept activated in the presence of sufficient Gal4. **b** Biomass and tocotrienols yield of YS-M5 under regulation of different temperature control systems in shake-flask cultures. All values presented are the means of three biological replicates, and error bars represent standard deviations. Source data are provided as a Source Data file.

To achieve high-density fermentation of tocotrienols, temperature-responsive regulation was adopted to enable two-stage fermentation. The wild-type Gal4 was knocked out from YS-M5, and the previously created temperature-sensitive Gal4 mutant Gal4M9^32^ was introduced under control of P_*ACT1*_, generating strain YS-M5T1 (YS-M5, ΔG*AL*4::*HIS3*, Δ*GAL80*::P_*ACT1*_-*GAL4M9*). As expected, separation of the growth stage and the tocotrienols production stage was observed in shake-flask cultures of YS-M5T1 with temperature shift, and higher biomass was achieved, but the yield of tocotrienols was very low, only 16% of YS-M5 (Fig. 7b). The low tocotrienols production in the Gal4M9-based temperature control system could be possibly ascribed to the low activity of Gal4M9 resulted from the trade-off between activity and temperature sensitivity during directed evolution^32^. Therefore, we tried to make up for the activity loss by expressing Gal4M9 under a strong promoter (P_*PGK1*_), constructing strain YS-M5T2 (YS-M5, ΔG*AL*4::*HIS3*, Δ*GAL80*::P_*PGK1*_-*GAL4M9*). The yield of tocotrienols was increased by 77% as compared to YS-M5T1, but the biomass decreased by 29%. Furthermore, obvious leakage of GAL4M9 expression at 30 °C was observed, and the yield of tocotrienols was only 28% that of YS-M5 (Fig. 7b).

To take advantage of temperature control and meanwhile to avoid the adverse effect of limited Gal4M9 activity on tocotrienols production, we redesigned the temperature control system by introducing an additional level of regulation for the P_*GAL*_-driven biosynthetic pathway (Fig. 7a). The wild-type Gal4 was placed under control of P_*GAL1*_ which could only be switched on by the P_*GAL4*_-driven Gal4M9. Upon depletion of glucose in the culture medium, P_*GAL4*_ initiates expression of the temperature-sensitive Gal4M9, which activates the expression of the P_*GAL1*_-driven Gal4 when the temperature is changed to 24 °C. The accumulated Gal4 can kick start the expression of P_*GAL*_-driven genes including its own encoding gene. In this way, a short-time incubation at 24 °C would be sufficient to switch on the target synthetic pathway. Considering that the accumulation of Gal4 under a strong *GAL* promoter may become excessive in this signal amplification system, which would impair cell growth^52,53^, the suitable promoter strength for proper control of the Gal4 amount was investigated. To weaken P_*GAL1*_ while maintaining its response to Gal4 binding, truncated P_*GAL1*_ variants were created by changing the number of Gal4 binding sites in UAS_G_, generating P_*GAL1*_-2 with 3 sites and P_*GAL1*_-3 with 2 sites^54^. Examination of the promoters in the lycopene-producing yeast Ylyc-TS0^32^ finalized P_*GAL1*_-3 as the one with appropriate strength to control Gal4 expression, and 5 h of 24 °C incubation was found sufficient to deliver temperature control (Supplementary Note 1).

Introduction of the cold-shock-triggered temperature control system into the tocotrienols high-producing strain YS-M5 generated strain YS-M5GP3 (YS-M5, Δ*GAL4*::*HIS3*, Δ*DPP1*::P_*GAL1*-3_-*GAL4*, Δ*GAL80*::P_*GAL4*_-*GAL4M9*). The tocotrienols yield of YS-M5GP3 in shake-flask cultures reached 5.2 mg/g DCW, which was 3.4 times higher than that of YS-M5T1 directly controlled by Gal4M9, and the biomass of YS-M5GP3 was higher than that of the glucose-regulated YS-M5 and similar to that of YS-M5T1 (Fig. 7b). These results demonstrated that the redesigned temperature control system was effective in relieving the metabolic burden of the tocotrienols-producing strain, and was meanwhile more efficient than the original temperature-responsive system.

### Temperature-regulated high-density fermentation

**Fig. 8:**
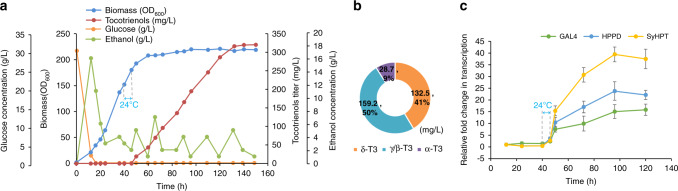
Temperature-regulated high-density fermentation of tocotrienols by YS-M5GP3(+). **a** Time courses of cell growth, tocotrienols production and consumption of carbon sources during temperature-regulated high-density fermentation of tocotrienols by YS-M5GP3(+). The duration of cold shock at 24 °C is from 40 to 45th h. **b** Composition of tocotrienols produced by the end of YS-M5GP3(+) fermentation. T3 is short for tocotrienol. **c** Transcription levels of *GAL4/GAL4M9*, *HPPD*, *SyHPT* in YS-M5GP3(+) during fermentation. All values presented are the means of three biological replicates, and error bars represent standard deviations. Source data are provided as a Source Data file.

For fed-batch fermentation, strain YS-M5GP3(+) was constructed by complementing the auxotrophic markers in YS-M5GP3. At the beginning of fermentation, the strain grew rapidly at 30 °C, and hardly any tocotrienols were produced. When the strain entered the mid-late-logarithmic growth phase at 40^th^ h, the culture temperature was changed to 24 °C to activate the temperature-sensitive Gal4M9 so as to kick start the accumulation of the wild-type Gal4. After 5 h of cold shock, the temperature was changed back to 30 °C until the end of the fermentation. At this stage, tocotrienols were accumulated due to the initiation of pathway gene expression upon binding of sufficient Gal4 to P_*GAL*_. Finally, the biomass reached to 220 OD_600_, and 320 mg/L of tocotrienols was produced after completion of fermentation at 150 h (Fig. 8a, b). Comparing to YS-M5(+) without temperature control, YS-M5GP3(+) produced 83% more tocotrienols.

To monitor the temperature response of the transcriptional activator and the pathway genes, the transcription levels of *GAL4/GAL4M9*, *HPPD* and *SyHPT* were monitored during the fermentation by real-time quantitative PCR (Fig. 8c). The transcriptional level of *GAL4/GAL4M9* was kept low and stayed stable before cold shock, excluding leakage of Gal4 expression at the restrictive temperature, which demonstrated the stringent control of this system. After cold shock, the transcription level of *GAL4/GAL4M9* began to increase gradually and reached a maximum of 15 folds, elucidating the successful switch-on of Gal4 expression, which continued even after the temperature was changed back to 30 °C. As expected, upregulation of the P_*GAL1*_-driven pathway genes *HPPD* and *SyHPT* (by 24 and 39 folds respectively) was also observed after cold shock.

## Discussion

Despite the numerous outstanding properties of tocotrienols, their synthesis is rarely studied. To make tocotrienols more readily available, we explored the fermentative production via constructing a tocotrienols-producing yeast cell factory.

First of all, genes were cloned from photosynthetic organisms and assembled in *S. cerevisiae* to construct a complete tocotrienols synthetic pathway together with the endogenous MVA pathway and shikimate pathway. During pathway construction, a major problem encountered was the difficulty in heterologous expression of HGGT in yeast. Among the total of 18 HGGT/HPT genes cloned from 14 photosynthetic organisms, only the SyHPT from *Synechocystis* was functionally expressed in yeast, whereas all HGGT/HPTs of plant sources failed to express. SyHPT is a bifunctional enzyme that can both synthesize the tocotrienol precursor MGGBQ with HGA and GGPP as substrates and generate the tocopherol precursor MPBQ with HGA and phytylpyrophosphate (PDP) as substrates^11^. Since there is no endogenous PDP-forming enzyme in yeast, SyHPT can be used to construct tocotrienols-producing yeast without concerns of forming tocopherols as byproducts. By introducing SyHPT together with the HPPD, MPBQMT, TC and γ-TMT from *A. thaliana*, a complete tocotrienols synthetic pathway was constructed in *S. cerevisiae*, leading to production of 313.0 μg/g DCW tocotrienols. As found in previous studies^55,56^, we were also unable to separate β-tocotrienol and γ-tocotrienol using HPLC. Although these two tocotrienol isomers could not be quantified respectively, γ-tocotrienol seemed to be the dominant component. When γ-TMT was introduced into YS-14, no change in δ-tocotrienol accumulation but obvious decrease in the peak corresponding to γ/β-tocotrienol together with production of α-tocotrienol was observed (Supplementary Fig. 4), indicating that γ-TMT had little catalytic activity for β-tocotrienol production from δ-tocotrienol in yeast. Further overexpression and truncation of γ-TMT also did not obviously decrease δ-tocotrienol accumulation. This result may be explained by the much higher substrate preference of the *A. thaliana*-derived γ-TMT for γ-tocotrienol as compared to δ-tocotrienol^57^.

To improve the tocotrienols production by the engineered yeast, the rate-limiting steps in the synthetic pathway were identified and eliminated by means of gene overexpression and protein truncation. Similar to natural producers^58^, the transfer of GGPP to HGA was found as a rate-limiting step in the tocotrienols synthetic pathway of yeast. Overexpression of SyHPT eliminated this bottleneck, leading to obvious accumulation of MGGBQ. To further pull the flux towards tocotrienols formation, MPBQMT, TC and γ-TMT were overexpressed in different combinations. However, significant amounts of MGGBQ remained unconverted, implying the presence of additional rate-limiting step that cannot be simply removed by protein overexpression. In plants, MPBQMT, TC and γ-TMT are all located on the inner membrane of chloroplasts, and their correct localization requires proper transit peptides^39,40^. When heterologously expressed in *S. cerevisiae* which lacks the mechanism to remove the plastid transit peptide, these peptides with misleading transit function may prevent proper protein expression^25^. By excising the predicted transit peptides from these proteins at proper sites, the catalytic activities of MPBQMT, TC and γ-TMT were all obviously improved. EGFP-reported expression of the full-length proteins and their variants created by truncating different lengths of transit peptide showed obvious enhancement of the fluorescence intensity after removal of the transit peptides, and the signal was positively correlated with the truncation length of the N-terminal sequence as well as the amount of DMGGBQ accumulation before the peak values were reached. The higher expression level of the truncated enzymes suggested that the heterologous expression of MPBQMT, TC and γ-TMT in yeast was indeed negatively influenced by the presence of the chloroplast transit peptide. For MPBQMT, its expression level reported by the fluorescence intensity was enhanced by only 1-fold, whereas its catalytic activity as judged by DMGGBQ accumulation increased by as much as 9 times. This result indicated that excision of the transit peptide also improved its catalytic activity. For the other two enzymes, excessive truncation of the N-terminal sequence (longer than 47 aa for TC and 40 aa for γ-TMT) led to reduced activity or even activity loss although the expression level was obviously higher than the intact proteins, implying an important role of the N-terminal residues in the catalysis. Surprisingly, the truncated TC and γ-TMT were inactivated when the top-scored transit peptides predicted through ChloroP 1.1 (98 aa and 50 aa, respectively) were removed, whereas excision of 47 aa and 40 aa transit peptides with relatively lower score led to enhanced protein expression. In addition, the transit peptides previously predicted for TC and γ-TMT (74 aa and 47 aa, respectively), which were removed before their functional expression in *E. coli*^57,59^, were not listed as high-score by the software we used. All these results suggested that the prediction results should be interpreted with caution, and more than one excision site with high CS score should be included in experimental verification.

Considering that the potential of the heterologous pathway may be limited by insufficient supply of endogenous precursors, the shikimate pathway and MVA pathway were both strengthened. As expected, comprehensive engineering of the shikimate pathway towards self-sufficient supply of 4-HPP led to higher HGA supply and tocotrienols production as compared to exogenous tyrosine addition, and enhancing GGPP supply further improved tocotrienols synthesis. To match with the reinforced precursor supply and restore metabolic balance, the heterologous pathway was further engineered to convert all accumulated intermediates to tocotrienols, with δ-tocotrienol accumulated as a major component at this stage. To drive more flux towards the methylated tocotrienol isomers (α- and γ- tocotrienol) which were reported with higher oral bioavailability^60^, stronger antioxidant activity^61^ than δ-tocotrienol, the catalytic capacity of the methyltransferases is crucial. Excessive overexpression of tMPBQMT under the strong P_*GAL10*_ resulted in decreased biomass and low yields, whereas proper enhancement of tMPBQMT expression driven by the medium-strength P_*IDI*_ led to elevated accumulation of the methylated tocotrienols. To further improve the catalytic activity of the methyltransferases, the supply of the methyl donor SAM was enhanced by SAM2 overexpression. As expected, higher yields of γ/β-tocotrienol and α-tocotrienol were obtained. Although overexpression of the *Arabidopsis* γ-TMT in plant seeds resulted in the near-complete conversion of γ-tocopherol to α-tocopherol^57,62^ and γ-tocotrienol to α-tocotrienol^15^, the activity of γ-TMT seemed to be low when expressed in yeast, with γ-tocotrienol remaining as the main product. After comprehensive optimization of the whole pathway, the tocotrienols yield was improved to 7.6 mg/g DCW. However, this strain failed to achieve high-density fermentation.

As reported for carotenoids biosynthesis, accumulation of lipophilic products in cell membrane often leads to growth inhibition^30^. Although the glucose-responsive regulation system based on *GAL80* deletion^31,63^ was adopted, the slow growth and low final biomass suggested failure of this system to relieve metabolic burden of these cells, possibly due to the leaky expression of the heterologous pathway at low glucose concentrations^32^. Accumulation of the target products prior to the planned switch-on time may impair cell growth. Taking into account that high cell density was achieved by glucose-regulated carotenogenic yeast^31,63^, tocotrienols accumulation may be more toxic to the yeast cells as compared to carotenoids. Considering the more stringent control of the temperature-responsive regulation system developed based on *GAL80* deletion and Gal4 engineering^32^, the wild-type Gal4 in the tocotrienols-producing yeast was replaced by the temperature-sensitive Gal4M9 to enable temperature-regulated two-stage fermentation. The biomass was indeed improved upon the complete separation of growth and production stages, however, the tocotrienols yield was largely decreased due to the lower activity of Gal4M9 as compared to the wild-type Gal4. Trade-off often occurs in protein engineering, with decrease of activity in return for gaining temperature sensitivity in this case. For proteins with known structure-function relationship, rational design could be conducted to compensate the trade-off^64^. However, the structure of Gal4 is only partially resolved^65^ and the molecular basis for its temperature sensitivity and activity remains unrevealed, discouraging its rational engineering. Therefore, we turned to expression regulation of Gal4M9 to make up for its activity loss. Simple overexpression by using a stronger promoter was proven to be an ineffective solution, with only slightly improved tocotrienols yield and lowered biomass.

A cold-shock-triggered temperature control system was then developed by using the temperature-sensitive Gal4M9 to control the expression of wild-type Gal4 so as to amplify the temperature-responsive regulation signal. Different from the traditional regulation systems where the pathway genes are directly regulated by the transcriptional factors, we placed the transcriptional activator Gal4 under control of its temperature-sensitive variant. In this way, the activity reduction of Gal4M9 could be compensated while maintaining its temperature sensitivity, leading to obvious improvement in the product yield. Moreover, a few hours’ incubation at the permissive temperature is sufficient in this system, making it more energy-saving than the original system where a low temperature should be maintained for the whole product accumulation stage. Although leaky expression of Gal4 as implied by the accumulation of tocotrienols at the restrictive temperature was observed in shake-flask culture, tocotrienols synthesis was stringently controlled as a two-stage process in fed-batch fermentation possibly due to the more precise control of fermentation parameters. The obviously improved biomass as compared to the glucose-responsive regulation system and much higher tocotrienols production than the original temperature-responsive regulation system demonstrated the stringency and efficiency of this upgraded temperature control system in two-stage fermentation. For further optimization of this system, artificial Gal4 binding sites may be created by directed evolution and used to fine-tune the expression level of the wild-type Gal4, so as to deliver custom-made regulation systems for two-stage fermentation of different pathways.

To sum up, a tocotrienols high-producing yeast strain was successfully constructed by adopting a cold-shock-triggered temperature control system in addition to comprehensive optimization of the heterologous pathway and the endogenous precursor pathways, enabling the fermentative production of tocotrienols with titers of up to 320 mg/L, which was two orders of magnitude higher than the recently reported δ-tocotrienol-producing yeast with titer of 4.1 mg/L^24^. The biosynthesis of γ- and α-tocotrienols is made possible in nonphotosynthetic organisms. This work would lay a solid foundation for fermentative production of vitamin E tocochromanols, and the cold-shock-triggered temperature control system developed would provide a powerful regulation tool for biosynthesis of natural products in yeast.

## Methods

### Strains cultivation

*E. coli* DH5α was used for plasmid propagation, and *S. cerevisiae* BY4741^66^ was employed as the host for yeast strain construction, with the details listed in Table 1. Yeast cells were cultivated in YPD medium, and 200 µg/mL geneticin (G418) was added as selection pressure for the recombinants containing *KanMX* marker. For the transformants with auxotroph markers, SD medium omitting the corresponding amino acids was used. SD complete medium with 1 mg/mL of 5-Fluoroorotic acid (5-FOA) was used for counter-selection of recombinants containing the *URA3* marker^66^. Strains producing homogentisic acid (HGA) were cultivated in SD complete medium. For exogenous supply of tyrosine, 0.1% (w/v) tyrosine was added to the medium before sterilization. For shake-flask culture, single colonies were picked from agar plate and inoculated into test tubes containing 5 mL of appropriate medium for overnight growth at 30 °C in a 220 rpm orbital shaker, then the precultures were inoculated into shake flasks containing 50 mL medium to an initial OD_600_ of 0.05^47^.

**Table 1:** Yeast strains used or constructed in this study.

Strain name	Genotype/description	Source
YS-01	BY4741, ΔG*AL80, HMG1*::T_*ADH1*_-*tHMG1*-P_*GAL10*_-P_*GAL1*_*-CrtE*-T_*CYC1*_	This study
YS-11	YS-01, Δ*HO*::P_*GAL1*_*-HPPD*-T_*CYC1*_	This study
YS-12	YS-11, Δ*DPP1*::P_*GAL1*_*-SyHPT*-T_*CYC1*_	This study
YS-13	YS-11, Δ*DPP1*::T_*ADH1*_-*MPBQMT*-P_*GAL10*_-P_*GAL1*_*-SyHPT*-T_*CYC1*_	This study
YS-14	YS-13, ΔG*AL1-7*::P_*GAL1*_-*TC*-T_*CYC1*_	This study
YS-15	YS-13, ΔG*AL1-7*::T_*ADH1*_-*γ-TMT-*P_*GAL10*_-P_*GAL1*_-*TC*-T_*CYC1*_	This study
YS-2	YS-15, ΔG*AL80*::P_*GAL1*_*-SyHPT*-T_*CYC1*_	This study
YS-23	YS-15, ΔG*AL80::*T_*ADH1*_-*MPBQMT-*P_*GAL10*_-P_*GAL1*_*-SyHPT*-T_*CYC1*_	This study
YS-24	YS-15, ΔG*AL80*::T_*ADH1*_-*TC-*P_*GAL10*_-P_*GAL1*_*-SyHPT*-T_*CYC1*_	This study
YS-25	YS-15, ΔG*AL80*::T_*ADH1*_-*γ-TMT-*P_*GAL10*_-P_*GAL1*_*-SyHPT*-T_*CYC1*_	This study
YS-234	YS-23, Δ*Ty4*::P_*GAL1*_-*TC*-T_*CYC1*_	This study
YS-2345	YS-23, Δ*Ty4*::T_*ADH1*_-*γ-TMT-*P_*GAL10*_-P_*GAL1*_*-TC*-T_*CYC1*_	This study
YS-15C	YS-11, Δ*DPP1*::T_*ADH1*_-*tMPBQMT-*P_*GAL10*_-P_*GAL1*_*-SyHPT*-T_*CYC1*_, Δ*GAL1-7*::T_*ADH1*_-*tTMT-*P_*GAL10*_*-*P_*GAL1*_*-tTC*-T_*CYC1*_	This study
YS-2C	YS-15C, ΔG*AL80*::P_*GAL1*_*-SyHPT*-T_*CYC1*_	This study
YS-23C	YS-15C, ΔG*AL80*::T_*ADH1*_-*tMPBQMT-*P_*GAL10*_-P_*GAL1*_*-SyHPT*-T_*CYC1*_	This study
YS-234C	YS-23C, Δ*Ty4*::P_*GAL1*_*-tTC*-T_*CYC1*_	This study
YS-24C	YS-15C, ΔG*AL80*::T_*ADH1*_-*tTC-*P_*GAL10*_-P_*GAL1*_*-SyHPT*-T_*CYC1*_	This study
YS-245C	YS-2C, Δ*Ty4*::T_*ADH1*_-*tTMT-*P_*GAL10*_-P_*GAL1*_*-tTC*-T_*CYC1*_	This study
YS-M1	YS-245C, Δ*ARO3*::T_*ADH1*_-*Aro7*^*G141S*^-P_*GAL10*_-P_*GAL1*_*-A*ro*4*^*k229L*^ -T_*CYC1*_, Δ*ARO10*::T_*ADH1*_-*TyrC-*P_*GAL10*_-P_*GAL1*_-*TKL1*-T_*CYC1*_	This study
YS-M3	YS-M1, Δ*YPL062W*::T_*ADH1*_-P_*GAL10*_*-CrtE03M*-T_*ADH1*_	This study
YS-M4	YS-M3, Δ*LPP1*::T_*ADH1*_-*tTMT-*P_*GAL10*_-P_*GAL1*_*-tTC*-T_*CYC1*_	This study
YS-M5	YS-M4, Δ*YPL062W*:: P_*GAL1*_*-SAM2*-T_*CYC1*_, Δ*Ty4:*:P_*IDI1*_*-tMPBQMT*-T_*PGK1*_	This study
YS-M5(+)	YS-M5, *met15*::*MET15*, *his3*::*HIS3*, *leu2*::*LEU2*	This study
YS-M5T0	YS-M5, ΔG*AL*4::*HIS3*	This study
YS-M5T1	YS-M5T0, ΔG*AL80*::P_*ACT1*_*-GAL4M9*-T_*ADH1*_	This study
YS-M5T2	YS-M5T0, ΔG*AL80*::P_*PGK1*_*-GAL4M9*-T_*PGK1*_	This study
YS-M5GP3	YS-M5T0, Δ*DPP1*::P_*GAL1*-3_-*GAL4*-T_*CYC1*_*;* ΔG*AL80*::P_*GAL4*_-*GAL4M9*-T_*PGK1*_	This study
YS-M5GP3(+)	YS-M5GP3, *met15*::*MET15*, *leu2*::*LEU2*	This study
YXWP-111	BY4741, ΔG*AL80*::*LEU2, Δho*::T_*TPS1*_*-tHMG1-*P_*GAL7*_*-*P_*GAL2*_*-crtYB11M-*T_*PGK1*_*-*T_*CYC1*_*-crtI-*P_*GAL1*_*-*P_*GAL10*_*-CrtE03M-*T_*ADH1*_	Ref.^47^
Ylyc-TS0	YXWP-111, ΔG*AL*4::*HIS3*	Ref.^32^
Ylyc-TS01	Ylyc-TS0, Δ*LPP1*::P_*ACT1*_-*GAL4M9*-T_*ADH1*_	
Ylyc-GP1	Ylyc-TS0, Δ*LPP1*::P_*GAL4*_-*GAL4M9*-T_*ADH1*_; *Ty4*::P_*GAL1*_-*GAL4*-T_*CYC1*_	This study
Ylyc-GP2	Ylyc-TS0, Δ*LPP1*::P_*GAL4*_-*GAL4M9*-T_*ADH1*_; *Ty4*::P_*GAL1*-2_-*GAL4*-T_*CYC1*_	This study
Ylyc-GP3	Ylyc-TS0, Δ*LPP1*::P_*GAL4*_-*GAL4M9*-T_*ADH1*_; *Ty4*::P_*GAL1*-3_-*GAL4*-T_*CYC1*_	This study

The media components were purchased from Angel yeast Co., Ltd (Yichang, China). The antibiotics and primers were purchased from Sangon (Shanghai, China). The restrictive enzymes, T4 DNA ligase, Prime STAR HS DNA polymerase and RNAiso Plus reagent were purchased from Takara (Dalian, China). Yeast DNA Kit and HP Plant RNA kit were purchased from Omega (Georgia, America). Standard of homogentisic acid was purchased from TCI (TCI Co., Ltd, Japan). Standards of α-, γ-, δ-, and β-tocotrienols were purchased from Solarbio (Beijing, China). Standard of lycopene was purchased from Sigma–Aldrich (St. Louis, MO, USA).

### Gene cloning and plasmid construction

The genes in the vitamin E synthesis pathway were cloned from plants and algae. For the genes from plants, mRNA was first extracted from plant tissues and then reverse-transcribed into cDNA, and the genes were amplified using the primers designed based on the known sequences derived from GenBank. The algal genes were cloned using the genome of *Synechocystis* sp. PCC6803 and the cDNA of *Chlamydomonas reinhardtii* as templates, respectively. Codon optimization of these genes was conducted on www.jcat.de, and the optimized sequences were sent for chemical synthesis at Jierui (Shanghai, China). The expression and integration vectors of the codon-optimized genes were constructed via digestion and ligation. The donors for gene editing via the CRISPR/Cas9 system were designed using CRISPy (http://staff.biosustain.dtu.dk/laeb/crispy_yeast/)^67^. Detailed information on the construction of yeast episomal and integration plasmids is shown in Supplementary Table 2. All the primers used in this study are listed in Supplementary Table 3. The flowchart of yeast strain construction is summarized in Supplementary Fig. 6.

### EGFP fusion and fluorescence detection

EGFP was fused to the C-terminus of the heterologous pathway enzymes by overlapping extension PCR to report their expression in *S. cerevisiae*. The fluorescence of the recombinants was observed under blue light excitation using a fluorescence microscope (Nikon Eclipse Ti, Japan). The localization of fluorescence protein was observed by laser confocal microscopy (Nikon A1, Japan). The fluorescence intensity was quantitatively analyzed using a microplate reader (Tecan Infinite 200 PRO, Switzerland) at 488 nm excitation wavelength and 509 nm emission wavelength after diluting the cell density to OD_600_ = 1.

### Prediction and excision of chloroplast transit peptides

The potential transit peptides of MPBQMT, TC and γ-TMT from *Arabidopsis thaliana* were predicted through ChloroP 1.1 (www.cbs.dtu.dk/services/ChloroP/)^68^, and the cleavage sites were determined according to the search result of chloroplast transit peptides (cTP) and cleavage site score (CS score). The potential transit peptides with high CS scores were excised by PCR-directed amplification.

### Fed-batch fermentation

After overnight cultivation in YPD medium at 30 °C, the seed cultures were inoculated (10%, v/v) into a 5 L bioreactor (Shanghai Baoxing Co., Ltd, China) with 2.5 L medium. The fermentation medium contained 15 g/L yeast extract, 25 g/L corn steep powder and 30 g/L glucose. The agitation speed was adjusted from 300 to 600 rpm with a constant air input flow rate of 2.0 vvm to keep the dissolved oxygen at 40% of air saturation, and 15% (w/v) ammonium hydroxide was automatically added to maintain the pH at 5.5. Feeding was conducted using a two-stage strategy. At the first stage, 1 L of feeding solution^69^ consisting of 500 g/L glucose, 9 g/L KH_2_PO_4_, 2.5 g/L MgSO_4_, 3.5 g/L K_2_SO_4_, 0.28 g/L Na_2_SO_4_, 10 mL/L trace metal solution, and 12 mL/L vitamin solution was used to sustain fast cell growth. At the second stage, when the strain entered the late-logarithmic growth phase, the feeding solution was changed to 400 g/L ethanol to support tocotrienols accumulation and the ethanol concentration in the fermentation broth was maintained below 5 g/L. In case of fermentation under cold-shock-triggered temperature control, the temperature was switched from 30 to 24 °C at the beginning of the second feeding stage, kept for 5 h, and then changed back to 30 °C.

### Extraction and analysis of products

For determination of HGA concentration, the yeast strains were cultivated in SD medium and the supernatant of the fermentation broth was sampled, since HGA was secreted into the medium. After centrifugation of the fermentation broth, 1 mL of the supernatant was collected and mixed with 40 μL of glacial acetic acid, and used for HPLC analysis. HGA was detected by HPLC (SHIMADZU LC-20AT, Japan) equipped with a C18-H column (4.6 × 250 mm, 5 µm, YMC-Pack ODS-AQ, Japan) using a UV/VIS detector at 290 nm. Samples were eluted with 0.01 M KH_2_PO_4_ solution (A) and methanol (B) at 90/10% with a flow rate of 0.8 mL/min at 40 °C^70^.

To extract tocotrienols and the metabolic intermediates, fermentation broth was collected from shaking flasks or the bioreactor and centrifuged. The cell pellet was washed twice with distilled water, resuspended in 200 μL of distilled water and transferred into a 2 mL centrifuge tube containing 500 μL grinding beads (Ф = 0.5 mm). The cells were disrupted in an automatic sample grinding machine (Shanghai Jingxin Co., Ltd, China). The cell lysates were then extracted twice with 1 mL of acetone. The extracts were analyzed by HPLC (SHIMADZU LC-20AT, Japan) equipped with a C18-H column (4.6 × 250 mm, 5 µm, Agilent, ZORBAX, SB-C18, America) using a UV/VIS detector at 292 nm^21^. The mobile phase consisted of acetonitrile (A) and pure water (B), and the samples were eluted using a gradient elution program with a flow rate of 0.8 mL/min at 40 °C: 0–10 min, linear gradient from 70% A/30% B to 90% A/10% B; 10–40 min, linear gradient from 90% A/10% B to 100% A/0% B; 40–70 min, 100% A/0% B; 70–71 min, linear gradient from 100% A/0% B to 70% A/30% B^21^. Because commercial MGGBQ and DMGGBQ standards were not available, and their characteristic groups are the same as tocotrienols in HPLC detection, we adopted the standard curves of δ-tocotrienol and γ-tocotrienol to calculate their yields, respectively, so as to enable the comparison of yield changes in the same products among different strains.

Lycopene was extracted from the HCl-heat-treated cells with acetone. Briefly, the cell pellet after washed twice was resuspended in 3 M HCl, heated in a boiled water bath for 3 min and then cooled in an ice-bath for 3 min, and resuspended in acetone and vortexed for 5 min^31^ and measured by HPLC (SHIMADZU LC-20AT, Japan). Samples were eluted on an Amethyst C18-H column (4.6 × 150 mm, 5 µm, Sepax Technologies, Inc., Newark, DE) using 50% acetonitrile and 50% methyl alcohol isopropyl alcohol (3:2, v/v) as the mobile phase. The flow rate was 1.0 mL/min, and the column temperature was 40 °C^71^. The signals were detected by a UV/VIS detector at 470 nm.

### Liquid chromatography-mass spectrometry analysis

Liquid chromatography-mass spectrometry (LC-MS) was performed with a Bruker amaZon ETD mass spectrometer (Bruker, Germany) equipped with an electrospray ionization (ESI) device. Mass fragmentation spectra of the extracted samples and standard compounds were monitored over a mass range (*m/z*) of 100–1000. The condition of LC was the same as that of HPLC. The MS parameters used were: dry gas flow rate of 8 L/min, dry gas temperature of 220 °C, and nebulizer pressure of 25 psi.

### Real-time quantitative PCR analysis

RNA was extracted using RNAiso Plus Kit (TaKaRa, Japan) following the manufacturer’s instructions. Genomic DNA elimination and reverse transcription were conducted using a PrimeScript™ RT reagent Kit with gDNA Eraser (TaKaRa, Japan). Quantitative PCR was performed on Eppendorf Mastercycler EP Realplex2 Real-time PCR system (Hamburg, Germany) with SYBR^®^ Premix Ex Taq™ II (Tli RNaseH Plus) (TaKaRa, Japan) and primers as listed in Supplementary Table 3. The relative changes in mRNA level expression were calculated using the 2^−ΔΔCT^ method^32,72^, with CT = threshold cycle, ΔCT = CT(target gene) − CT(*ACT1*), and ΔΔCT = ΔCT(experimental) − ΔCT(reference). The ΔCT values of the sample taken before temperature shift (at 12 h) were used as reference here.

### Reporting summary

Further information on research design is available in the Nature Research Reporting Summary linked to this article.

## Supplementary information

Supplementary Information

Peer Review

Reporting summary

## Data Availability

Data supporting the findings of this work are available within the paper and its Supplementary Information files. The datasets and materials generated and analyzed during the current study are available from the corresponding author upon request. A reporting summary for this Article is available as a Supplementary Information file. The codon-optimized sequences used in this study are available in figshare [https://figshare.com/articles/dataset/Codon_optimized_sequence/12951011/1]. Source data are provided with this paper.

## Source data

Source Data
