# Supplementary material for: Fermentative production of Vitamin E tocotrienols in Saccharomyces cerevisiae under cold-shock-triggered temperature control
Source: Nat Commun. 2020 Oct 14;11:5155. doi: 10.1038/s41467-020-18958-9 (PMC7560618; doi:10.1038/s41467-020-18958-9)
Supplement: Supplementary file 1 — Supplementary Information [file 41467_2020_18958_MOESM1_ESM.pdf]

Fermentative production of Vitamin E tocotrienols in *Saccharomyces cerevisiae* under cold-shock-triggered temperature control

Shen *et al.*

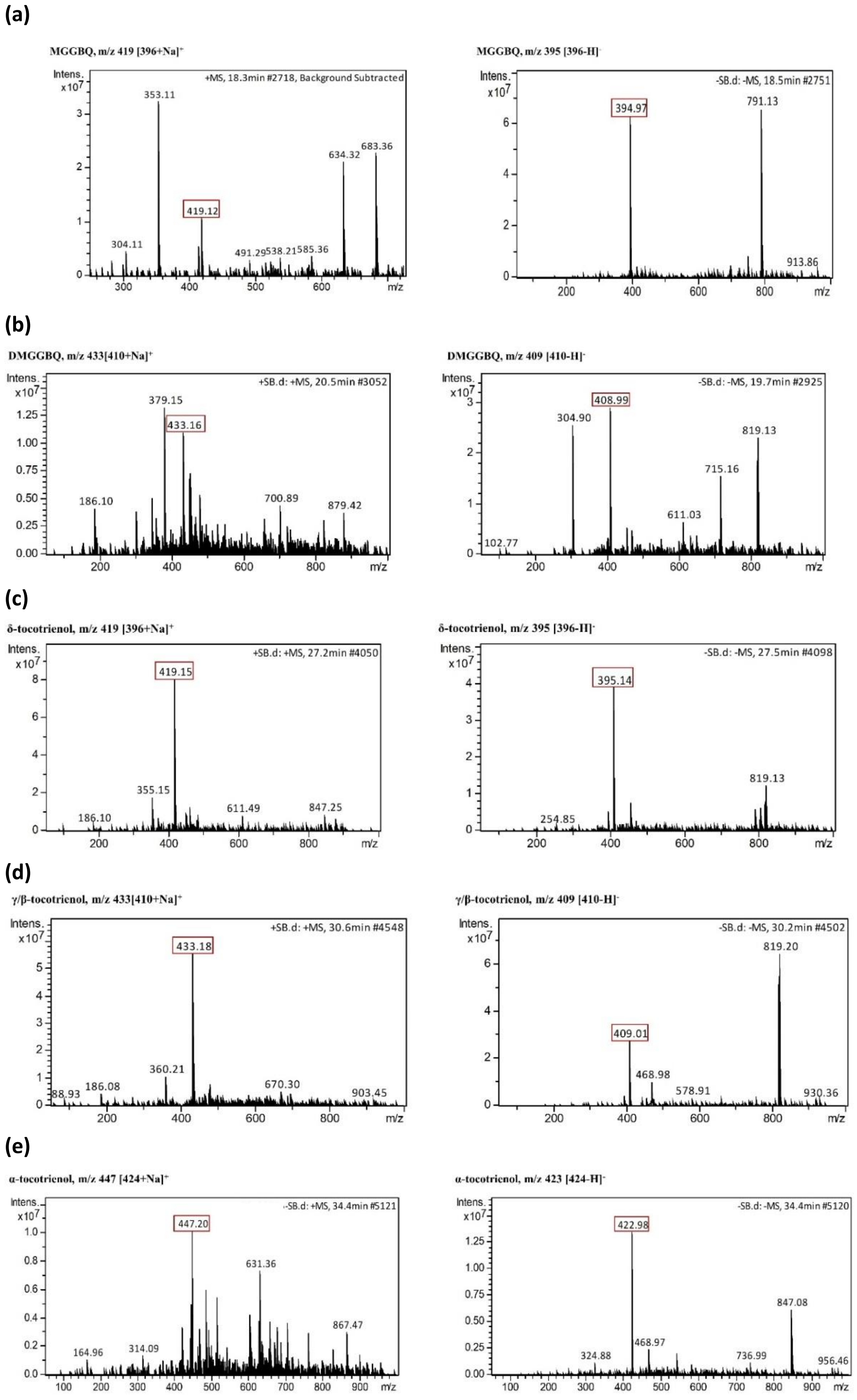

Supplementary Fig. 1. LC-MS analysis of tocotrienols and their precursors. a, MGGBQ; b, DMGGBQ; c, δ-tocotrienol; d, γ/β-tocotrienol; e, α-tocotrienol.

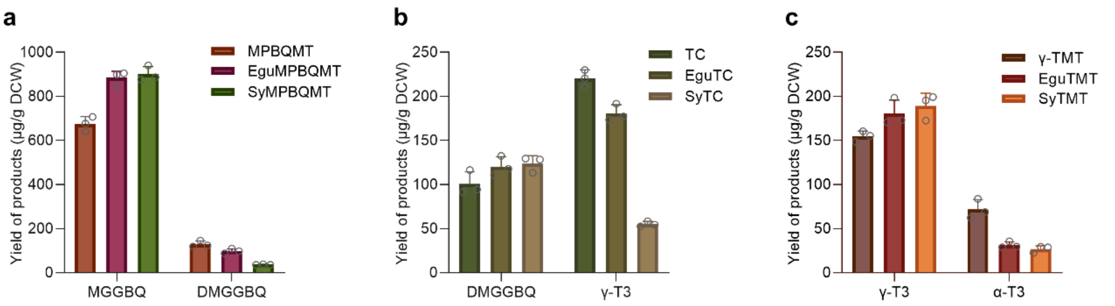

Supplementary Fig. 2. Comparison of catalytic activities of MPBQMT, TC and TMT from different organisms when expressed in yeast as shown by the variations in the corresponding substrate and product. a, MPBQMT; b, TC; c, TMT. Different colors represent enzymes from different sources, names without prefix refer to those from *Arabidopsis thaliana*, names prefixed with "Egu" refer to those from *Elaeis guineensis*, and names prefixed with "Sy" refer to those from *Synechocystis* sp. PCC6803. All values presented are the means of three biological replicates, and the error bars represent standard deviations. Source data are provided as a Source Data file.

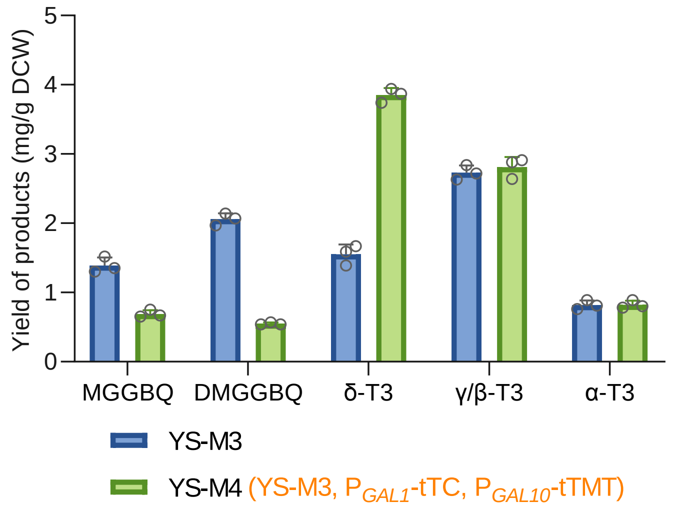

Supplementary Fig. 3. Comparison of metabolites production by YS-M3 and YS-M4. All values presented are the means of three biological replicates, and the error bars represent standard deviations. Source data are provided as a Source Data file.

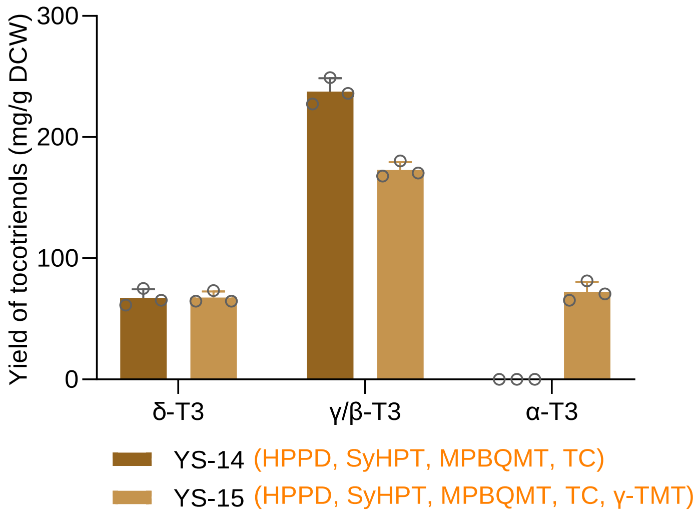

Supplementary Fig. 4. Comparison of tocotrienols production by YS-14 and YS-15. All values presented are the means of three biological replicates, and the error bars represent standard deviations. Source data are provided as a Source Data file.

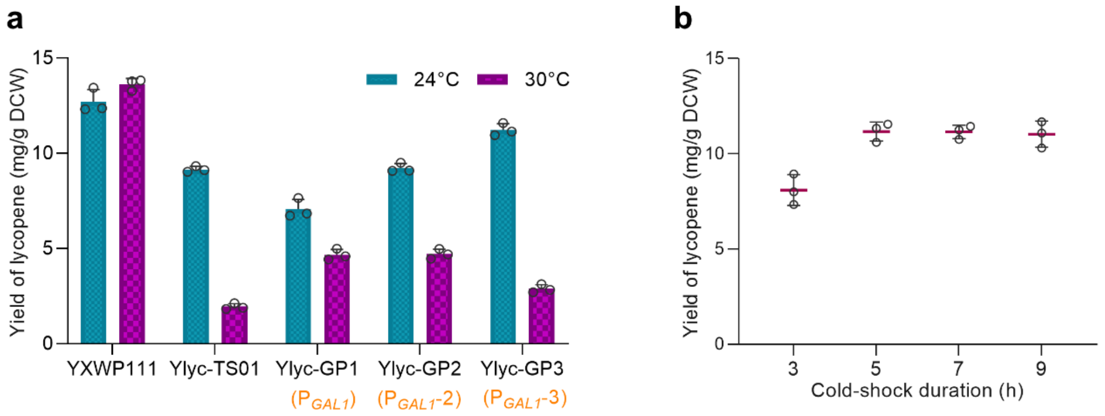

Supplementary Fig. 5. Examination of the cold-shock-triggered temperature control system in the lycopene-producing yeast. (a) Optimization of the cold-shock-triggered temperature control system based on the lycopene-producing yeast Ylyc-TS0. Modified P_{GAL1} with appropriate strength was screened to control the expression of the wild-type Gal4. **(b)** Determination of the suitable duration of cold shock in Ylyc-GP3. All values presented are the means of three biological replicates, and error bars represent standard deviations. Source data are provided as a Source Data file.

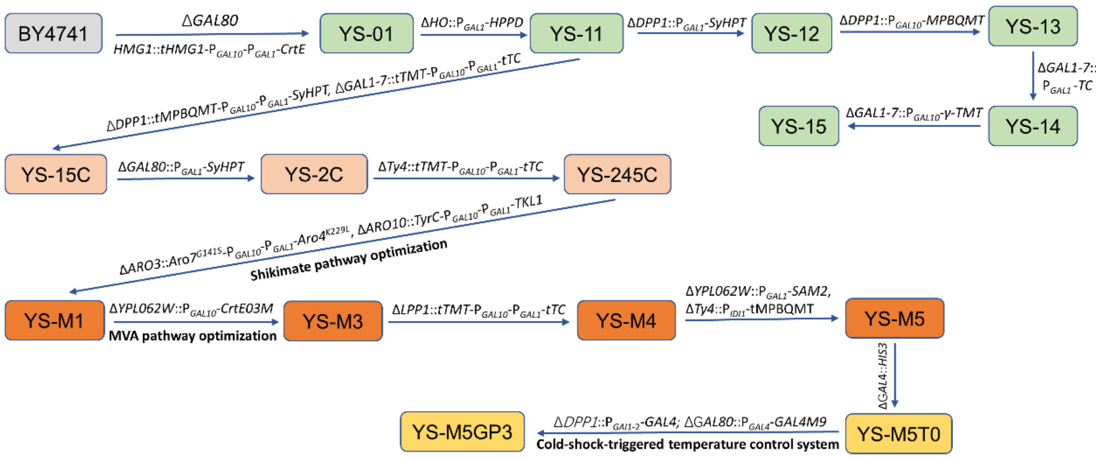

Supplementary Fig. 6. Flowchart of yeast strain construction in this study.

Supplementary Table 1. HPT/HGGT genes cloned from photosynthetic organisms.

Source	Name	NCBI Accession NO.
Arabidopsis thaliana	AtHPT1	NM_179653.4
	AtHPT2	DQ231060.1
Nicotiana tabacum	NiHPT	XM_016579562.1
***Synechocystis* sp. PCC 6803**	SyHPT	BAA17774
Chlamydomonas reinhardtii	CrHPT	LM999966.1
Triticum aestivum	TaHGGT	DQ231056.1
Hordeum vulgare	HvHGGT	AY222860.1
Zea mays	ZmHPT	NM_001112407.1
Oryza sativa japonica	OsHPT	XM_015786513.2
	OsHGGT	XM_015789025.2
Glycine max	GmHPT1	DQ231059.1
	GmHPT2	NM_001251443.2
Sesamum indicum	SiHPT	NM_001319684.1
Cucumis sativus	CsHPT	NM_001319684.1
Capsicum annuum	CaHPT	XM_016694630.1
Lactuca sativa	LsHPT	FJ194492.1
Elaeis guineensis	EguHPT	NP_001291355
	EguHGGT	XP_010932231

Supplementary Table 2. Plasmids used or constructed in this study.

Plasmid name	Genotype/ Description	Reference
pUMRI-10	*loxp-KanMX-URA3-pbr322ori-loxp*, T$_{ADH1}$-*MCS1*-P$_{GAL10}$-P$_{GAL1}$-*MCS2*-T$_{CYC1}$, *HO* homologous arm	GenBank: KM216412
pUMRI-11	*loxp-KanMX-URA3-pbr322ori-loxp*, T$_{ADH1}$-*MCS1*-P$_{GAL10}$-P$_{GAL1}$-*MCS2*-T$_{CYC1}$, *DPP1* homologous arm	GenBank: KM216413
pUMRI-13	*loxp-KanMX-URA3-pbr322ori-loxp*, T$_{ADH1}$-*MCS1*-P$_{GAL10}$-P$_{GAL1}$-*MCS2*-T$_{CYC1}$, *GAL1-7* homologous arm	GenBank: KM216415
pESC-URA	*f1 ori-URA3-2μ-AmpR-ori,* T$_{ADH1}$-*MCS1*-P$_{GAL10}$-P$_{GAL1}$-*MCS2*-T$_{CYC1}$	GenBank: AF063585
PMRI-21-*CrtE-tHMG1*	*loxp-KanMX-pbr322ori-loxp,* T$_{ADH1}$-*CrtE*-P$_{GAL10}$-P$_{GAL1}$-*tHMG1*-T$_{CYC1}$, *HMG1* homologous arm	This study
pUMRI-16	*loxp-KanMX-URA3-pbr322ori-loxp*, T$_{ADH1}$-*MCS1*-P$_{GAL10}$-P$_{GAL1}$-*MCS2*-T$_{CYC1}$, *LPP1* homologous arm	This study
pUMRI-17	*loxp-KanMX-URA3-pbr322ori-loxp,* T$_{ADH1}$-*MCS1*-P$_{GAL10}$-P$_{GAL1}$-*MCS2*-T$_{CYC1,}$ *Ty4* homologous arm	This study
pUMRI-18	*loxp-KanMX-URA3-pbr322ori-loxp,* T$_{ADH1}$-*MCS1*-P$_{GAL10}$-P$_{GAL1}$-*MCS2*-T$_{CYC1}$, *GAL80* homologous arm	This study
pUMRI-10-*HPPD*	pUMRI-10, T$_{ADH1}$-MCS1-P$_{GAL10}$-P$_{GAL1}$-*HPPD*-T$_{CYC1}$	This study
pUMRI-11-*SyHPT*	pUMRI-11, T$_{ADH1}$-MCS1-P$_{GAL10}$-P$_{GAL1}$-*SyHPT*-T$_{CYC1}$	This study
pUMRI-11-*SyHPT-MPBQMT*	pUMRI-11, T$_{ADH1}$-*MPBQMT*-P$_{GAL10}$-P$_{GAL1}$-*SyHPT*-T$_{CYC1}$	This study
pUMRI-13-*TC*	pUMRI-13, T$_{ADH1}$-MCS1-P$_{GAL10}$-P$_{GAL1}$-*TC*-T$_{CYC1}$	This study
pUMRI-13-*TC-γ-TMT*	pUMRI-13, T$_{ADH1}$-*γ-TMT*-P$_{GAL10}$-P$_{GAL1}$-*TC*-T$_{CYC1}$	This study
pUMRI-18-*HPPD*	pUMRI-18, T$_{ADH1}$-MCS1-P$_{GAL10}$-P$_{GAL1}$-*HPPD*-T$_{CYC1}$	This study
pUMRI-18-SyHPT	pUMRI-18, T$_{ADH1}$-MCS1-P$_{GAL10}$-P$_{GAL1}$-*SyHPT*-T$_{CYC1}$	This study
pUMRI-18- *MPBQMT*	pUMRI-18, T$_{ADH1}$-*MPBQMT*-P$_{GAL10}$-P$_{GAL1}$-MCS1-T$_{CYC1}$	This study
pUMRI-18-*TC*	pUMRI-18, T$_{ADH1}$-MCS1-P$_{GAL10}$-P$_{GAL1}$-*TC*-T$_{CYC1}$	This study
pUMRI-18 -*γ-TMT*	pUMRI-18, T$_{ADH1}$-*γ-TMT*-P$_{GAL10}$-P$_{GAL1}$-MCS1-T$_{CYC1}$	This study
pUMRI-18-*SyHPT-MPBQMT*	pUMRI-18, T$_{ADH1}$-*MPBQMT*-P$_{GAL10}$-P$_{GAL1}$-*SyHPT*-T$_{CYC1}$	This study
pUMRI-18-*SyHPT-TC*	pUMRI-18, T$_{ADH1}$-*TC*-P$_{GAL10}$-P$_{GAL1}$-*SyHPT*-T$_{CYC1}$	This study

pUMRI-18-*SyHPT-γ-TMT*	pUMRI-18, T_{ADH1}-*γ-TMT*-P_{GAL10}-P_{GAL1}-*SyHPT*-T_{CYC1}	This study
pUMRI-17-*TC*	pUMRI-17, T_{ADH1}-MCS1-P_{GAL10}-P_{GAL1}-*TC*-T_{CYC1}	This study
pUMRI-17-*TC-γ-TMT*	pUMRI-17, T_{ADH1}-*γ-TMT* -P_{GAL10}-P_{GAL1}-*TC*-T_{CYC1}	This study
pUMRI-11-*SyHPT-tMPBQMT*	pUMRI-11, T_{ADH1}-*tMPBQMT*-P_{GAL10}-P_{GAL1}-*SyHPT*-T_{CYC1}	This study
pUMRI-13-*tTC*	pUMRI-13, T_{ADH1}-MCS1-P_{GAL10}-P_{GAL1}-*tTC*-T_{CYC1}	This study
pUMRI-13-*tTC- tTMT*	pUMRI-13, T_{ADH1}-*tTMT*-P_{GAL10}-P_{GAL1}-*tTC*-T_{CYC1}	This study
pUMRI-13-*TC- tTMT*	pUMRI-13, T_{ADH1}-*tTMT*-P_{GAL10}-P_{GAL1}-*TC*-T_{CYC1}	This study
pUMRI-18-*SyHPT-tMPBQMT*	pUMRI-18, T_{ADH1}-*tMPBQMT*-P_{GAL10}-P_{GAL1}-*SyHPT*-T_{CYC1}	This study
pUMRI-18-*SyHPT-tTC*	pUMRI-18, T_{ADH1}-*tTC*-P_{GAL10}-P_{GAL1}-*SyHPT*-T_{CYC1}	This study
pUMRI-18-*SyHPT-tTMT*	pUMRI-18, T_{ADH1}-*tTMT*-P_{GAL10}-P_{GAL1}-*SyHPT*-T_{CYC1}	This study
pUMRI-17-t*TC*	pUMRI-17, T_{ADH1}-MCS1-P_{GAL10}-P_{GAL1}-*tTC*-T_{CYC1}	This study
pUMRI-17-t*TC-tTMT*	pUMRI-17, T_{ADH1}-*tTMT* -P_{GAL10}-P_{GAL1}-*tTC*-T_{CYC1}	This study
pUMRI-21	*loxp-KanMX-URA3-pbr322ori-loxp*, T_{ADH1}-*MCS1*-P_{GAL10}-P_{GAL1}-*MCS2*-T_{CYC1}	GenBank；KM216411
pUMRI-ARO3	*loxp-KanMX-URA3-pbr322ori-loxp,* T_{ADH1}-*MCS1*-P_{GAL10}-P_{GAL1}-*MCS2*-T_{CYC1}, *ARO3* homologous arm	This study
pUMRI-ARO10	*loxp-KanMX-URA3-pbr322ori-loxp*, T_{ADH1}-*MCS1*-P_{GAL10}-P_{GAL1}-*MCS2*-T_{CYC1}, *ARO10* homologous arm	This study
pUMRI-YPL062W	*loxp-KanMX-URA3-pbr322ori-loxp*, T_{ADH1}-*MCS1*-P_{GAL10}-P_{GAL1}-*MCS2*-T_{CYC1}, *YPL062W* homologous arm	This study
pUMRI-ARO3-*Aro4*	pUMRI-ARO3, T_{ADH1}-*MCS1*-P_{GAL10}-P_{GAL1}-$Aro4^{K229L}$-T_{CYC1}	This study
pUMRI-ARO3-*Aro4-Aro7*	pUMRI-ARO3, T_{ADH1}-$Aro7^{G141S}$-P_{GAL10}-P_{GAL1}-$Aro4^{K229L}$-T_{CYC1}	This study
pUMRI-AR010-*TKL1*	pUMRI-ARO10, T_{ADH1}-*TKL1*-P_{GAL10}-P_{GAL1}-MCS2-T_{CYC1}	This study
pUMRI-ARO10-*TyrC*	pUMRI-Aro10, T_{ADH1}-MCS1-P_{GAL10}-P_{GAL1}-*TyrC*-T_{CYC1}	This study
pUMRI-ARO10-*TKL1-TyrC*	pUMRI-Aro10, T_{ADH1}-*TKL1*-P_{GAL10}-P_{GAL1}-*TyrC*-T_{CYC1}	This study
pUMRI-YPL062W-*CrtE03M*	pUMRI-YPL062W, T_{ADH1}-*CrtE03M*-P_{GAL10}-P_{GAL1}-MCS2-T_{CYC1}	This study
pUMRI-YPL062W-*SAM2*	pUMRI-YPL062W, T_{ADH1}-MCS1-P_{GAL10}-P_{GAL1}-*SAM2*-T_{CYC1}	This study
pUMRI-YPL062W-*CrtE03M-SAM2*	pUMRI-YPL062W, T_{ADH1}-*CrtE03M*-P_{GAL10}-P_{GAL1}-*SAM2*-T_{CYC1}	This study
pUMRI-16-c*TC-cTMT*	pUMRI-16, T_{ADH1}-*cTMT* -P_{GAL10}-P_{GAL1}-*cTC*-T_{CYC1}	This study
pUMRI-16-*GAL4M9*	pUMRI-16, P_{GAL4}-*GAL4M9*-T_{ADH1}	This study
pUMRI-17-P1	pUMRI-17, P_{GAl1}-*GAL4*-T_{CYC1}	This study

pUMRI-17-P2	pUMRI-17, $P_{GAl1\text{-}2}$-*GAL4*-T_{CYC1}	This study
pUMRI-17-P3	pUMRI-17, $P_{GAl1\text{-}3}$-*GAL4*-T_{CYC1}	This study
p416-p*TEF1*-Cas9-t*CYC1*	CEN/ARS, URA3, P_{TEF1}-Cas9-T_{CYC1}	This study
pESC-LEU-M3-*Ty4*	*f1 ori-LEU-2μ-AmpR-ori,*<br>*SNR52p-gRNA-Ty4-SUP4t*	This study
pESC-LEU-M3-*DPP1-GAL80*	*f1 ori-LEU-2μ-AmpR-ori,*<br>*SNR52p-gRNA-DPP1-SUP4t-*<br>*SNR52p-gRNA-GAL80-SUP4t*	This study

Supplementary Table 3. Primers used in this study.

Primer name	Sequence (5'-3')	Description
HPPD, BamHI-F	GCTGAGGATCCATGTGTTTGTCTTTGGCTTC	For amplification of key genes in tocotrienols synthetic pathway
HPPD, SalI-R	GCACGTCGACTTAACCAACCAATTGCTTAGC	
SyHPT, BamH-F	GCTGAGGATCCATGGCTACTATTCAAGCTTTTTGG	
SyHPT, SalI-R	GCACGTCGACTTAGAAAATAGTATTAGAAAAATT	
MPBQMT, EcoRI-F	GCTGAGAATTCATGGCTTCTTTGATGTTGAAC	
MPBQMT, NotI-R	TGATCGCGGCCGCTTAGATTGGTTGGTCCTTTGG	
TC, BamHI-F	GCTGAGGATCCATGGAAATCAGATCTTTGATCG	
TC, SalI-R	GCACGTCGACTTACAAACCTGGTGGCTTGAA	
TMT, NotI-F	GCATCGCGGCCGCATGAAGGCTACTTTGGCTG	
TMT, SpeI-R	CGTGAACTAGTTTACAATGGCTTTTGACAAGT	
Egu-TC-BamHI-F	GTCAGGATCCATGGACGCCGCCTTCGCTCT	
Egu-TC-SalI-R	GTACGTCGACCTAAAGACCAGGAGGCCTTAAA	
Egu-MPBQ-NotI-F	TATGCGGCCGCATGGCTTCCTCGGTGCTTAA	
Egu-MPBQ-SpeI-R	CTGAACTAGTTCATATTGGCATTCCCTTGGG	
Egu-TMT-NotI-F	TATGCGGCCGCATGACCTCTCTCCTCCACAC	
Egu-TMT-SpeI-R	CTGAACTAGTCTACGTAGGTTTACGACAGGC	
Sy-MPBQMT-NotI-F	GATCGCGGCCGCATGCCCGAGTATTTGCTTCT	
Sy-MPBQMT-SpeI-F	CTGAACTAGTTTAAGCTTGAGTGGCGTTAAG	
Sy-TC-BamHI-F	GTCAGGATCCATGAAATTTCCGCCCCACAG	
Sy-TC-SalI-R	GTACGTCGACTCAGAATGGCACTGTTTTTTTGC	
Sy-TMT-NotI-F	GATCGCGGCCGCATGGTTTACCATGTTAGGCC	
Syn-TMT-SpeI-R	CTGAACTAGTTCAAACTAAAGGCTTTATCCCCG	
HPPD, EGFP-R	CTTTAGACATAGAACCACCACCACCAACCAATTGCTTAGC	For construction of EGFP fusion proteins for the pathway enzymes
SyHPT, EGFP-R	CTTTAGACATAGAACCACCACCGAAAATAGTATTAGAAAAATT	
MPBQMT, EGFP-R	CTTTAGACATAGAACCACCACCGATTGGTTGGTCCTTTGG	
TC, EGFP-R	CTTTAGACATAGAACCACCACCCAAACCTGGTGGCTTGAA	
TMT, EGFP-R	CTTTAGACATAGAACCACCACCCAATGGCTTTTGACAAGT	
EGFP-F	GGTGGTGGTTCTATGTCTAAAG	
EGFP-SalI-R	GCATCGTCGACTTATTTGTACAATTCATCCAT	
EGFP-NotI-R	CGTGAACTAGTTTATTTGTACAATTCATCCAT	
MPBQ-51aa-EcoRI-F	CCGGAATTCATGTCTTCTTCTGTTTCTTCTTCT	For excision of chloroplast transit peptide with different lengths from MPBQMT, TC and γ-TMT
TC-41aa-BamHI-F	CGCGGATCCATGTCTATCTCTAGAGTTTCTGC	
TC-47aa-BamHI-F	CGCGGATCCATGGCTTCTATCTCTACTCCAAAC	
TC-68aa-BamHI-F	CGCGGATCCATGACTTCTCCAAACAGAGAATT	
TC-76aa-BamHI-F	CGCGGATCCATGACTCCACACTCTGGTTACCA	
TC-98aa-BamHI-F	CGCGGATCCATGGTTTCTATCCCAGAAAAGAGAG	
TMT-35aa-NotI-F	ATTTGCGGCCGCATGTCTTCTTCTGTTTCTATGACT	
TMT-40aa-NotI-F	ATTTGCGGCCGCATGACTACTACTAGAGGTAACG	
TMT-50aa-NotI-F	ATTTGCGGCCGCATGGCTGCTGCTGCTACTTC	
Aro3-upF	AAAGCTGGAGCTGGCCTTGTACCTTTGGCTGAAATTGGAA	For construction of

Aro3-upR	TTACGGGCCTTTATGGCCTGCGTATCTTCTCAAATTTA	pUMRI-Aro3
Aro3-DnF	ACGCAGGCCATAAAGGCCCGTAAAGAATTTTGCCAACA	
Aro3-DnR	TAATAGCGAAGAGGCCTACAATATTCCCAAAGAAGGTGGC	
P21-F1	TGTAGGCCTCTTCGCTATTACGCCA	
p21-R1	ACAAGGCCAGCTCCAGCTTTTGTT	
Aro10-UpF	AAAGCTGGAGCTGGCCTTGTAACCTATTACATACCGACAC	For construction of pUMRI-Aro10
Aro10-UpR	TATAAGGCCCATGAGGCCGCGAACCCCTTTTTTTCTTC	
Aro10-DnF	TTCGCGGCCTCATGGGCCTTATAGTTCTTCAAGTGGTG	
Aro10-DnR	CGTAATAGCGAAGAGGCCTACATCATTCAATGTCCCTCTAAA	
YPL062w-F	AAAGCTGGAGCTGGCCTTGTCACCGACCATGTGGGCAAAT	For construction of pUMRI-YPL062w
YPL062w-R	TAATAGCGAAGAGGCCTACAGCCCTTACGTGAGGGGCAGT	
YPL062wUp-F	CAACAAGTTTATGAAAGCTCGGCCATAAAGGCCACTACCACTG CACCTCCTAA	
YPL062wDn-R	TTAGGAGGTGCAGTGGTAGTGGCCTTTATGGCCGAGCTTTCATA AACTTGTTG	
Aro4-BamHI-F	CGGGATCCCGATGAGTGAATCTCCAATGTTCG	For creation of Aro4^{K229L} mutant
Aro4-SalI-R	ACGCGTCGACCTATTTCTTGTTAACTTCTCTTCTTTG	
Aro4-K229L-DnF	CATTTCATGGGTGTTACTTTGCATGG	
Aro4-K229L-UPR	GATAGCAGCAACACCATGCAAAGTAAC	
Aro7-NotI-F	GAATGCGGCCGCATGGATTTCACAAAACCAGAAAC	For creation of Aro7^{G141S} mutant
Aro7-SpeI-R	CTAGACTAGTTTACTCTTCCAACCTTCTTAGC	
Aro7-G141S-DnF	GATGATAAGAATAACTTCTCTTCTGTTG	
Aro7-G141S-upR	ATCTCTAGTGGCAACAGAAGAGAAGTT	
TKL1-BamHI-F	CGGGATCCCGATGACTCAATTCACTGACATTG	For amplification of *TKL1*, *TyrC*, *SAM2* and *CrtE03M*
TKL1-SalI-R	ACGCGTCGACTTAGAAAGCTTTTTTCAAAGGAG	
TyrC-NotI-F	GAATGCGGCCGCATGACAGTTTTTAAACATATTG	
TyrC-SpeI-R	CTAGACTAGTTTATGGATGAATATCATGATCT	
SAM2-BamHI-F	CGGGATCCCGATGTCCAAGAGCAAAACTTTC	
SAM2-SalI-R	ACGCGTCGACTTAAAATTCCAATTTCTTTGG	
CrtE03M-NotI-F	GAATGCGGCCGCATGGATTACGCGAACATCCTC	
CrtE03M-SpeI-R	CTAGACTAGTTCACAGAGGGATATCGGCT	
Pgal4-BamHI-F	TACGGATCCGACAGCATTCGCCCAGTATTT	For amplification of P_{GAL4}, *GAL4*, *GAL4M9*
Pgal4-EcoRI-R	GTTGAATTCCTTTCAGGAGGCTTGCTTCTC	
GAL4M9-NotI-F	ATTGCGGCCGCATGAAGCTACTGTCTTCTAT	
GAL4M9-SacI-R	TTAAGAGCTCTTACTCTTTTTTTGGGTTTGGTG	
GAL4-BamHI-F	CGCGGATCCATGAAGCTACTGTCTTCTATCG	
GAL4-NheI-R	CTAGCTAGCTTACTCTTTTTTTGGGTTTGG	
Pgal1-1-NotI-F	ATTTGCGGCCGCCGGATTAGAAGCCGCCGAGCGGGT	For amplification of P_{GAL1}, P_{GAL1}-2, P_{GAL1}-3
Pgal1-2-NotI-F	ATTTGCGGCCGCCGGGTGACAGCCCTCCGAAGGAAG	
Pgal1-3-NotI-F	ATTTGCGGCCGCAGGAAGACTCTCCTCCGTGCGTCC	
gRNA-Ty4-F	GCAGTGAAAGATAAATGATCTTAATATAATCGTATAAGAG	For construction of gRNA (*Ty4*, *GAL80*,
gRNA-Ty4-R	GCTATTTCTAGCTCTAAAACCTCTTATACGATTATATTAA	

gRNA-GAL80-F	GCAGTGAAAGATAAATGATCAATGCAGCTCCCATAAGAGT	*DPP1*) plasmids for the CRISPR/Cas9 system
gRNA-GAL80-R	CTATTTCTAGCTCTAAAACACTCTTATGGGAGCTGCATT	
gRNA-DPP1-F	GCAGTGAAAGATAAATGATCGGGTTTAGGACAACTCCGTC	

Supplementary Note 1. Examination of the cold-shock-triggered temperature control system in the lycopene-producing yeast

P_{GAL1} is a strong promoter with four Gal4 binding sites in the upstream activating sequence (UAS_G)[1], P_{GAL1} variants were constructed by changing the number of Gal4 binding sites in UAS_G, creating P_{GAL1}-2 with 3 sites and P_{GAL1}-3 with 2 sites[2]. For direct comparison of the promoter strength and its effect on the temperature-control system, the previously constructed lycopene-producing yeast Ylyc-TS0 was used as the host[3]. P_{GAL4}-driven Gal4M9 and Gal4 under control of P_{GAL1}, P_{GAl1}-2 and P_{GAl1}-3 respectively were introduced together into Ylyc-TS0 to construct strains Ylyc-GP1 (Ylyc-TS0, Δ*LPP1*::P_{GAL4}-*GAL4M9*; Δ*Ty4*::P_{GAl1}-*GAL4*), Ylyc-GP2 (Ylyc-TS0, Δ*LPP1::*P_{GAL4}-*GAL4M9*; Δ*Ty4*::$P_{GAl1\text{-}2}$-*GAL4*) and Ylyc-GP3 (Ylyc-TS0, Δ*LPP1*::P_{GAL4}-*GAL4M9*; Δ*Ty4*::$P_{GAl1\text{-}3}$-*GAL4*). After growing at 30°C to the late logarithmic phase, the culture temperature was lowered to 24°C and kept for 5 hours before changing back to 30°C, the lycopene yield of Ylyc-GP3 with P_{GAL1}-3-driven Gal4 was the highest (Supplementary Fig. 5a), which was close to that of YXWP111 harboring the wild-type Gal4 continuously cultured at 30°C[4], and 123% as much as that of Ylyc-TS01 controlled by the original Gal4M9-mediated temperature control system[3]. In order to determine the proper duration of 24°C incubation, Ylyc-GP3 was subjected to 3, 5, 7 and 9 hours of cold shock respectively during cultivation and 5 h cold shock was found to be enough (Supplementary Fig. 5b).

Supplementary References

1. West, R. W., Yocum, R. R., & Ptashne, M. *Saccharomyces cerevisiae GAL1-GAL10* divergent promoter region: location and function of the upstream activating sequence UAS_G. *Mol. Cell. Biol.* **4**, 2467-2478 (1984).

2. Giniger, E., Varnum, S. M., & Ptashne, M. Specific DNA binding of GAL4, a positive regulatory protein of yeast. *Cell* **40**, 767-774 (1985).

3. Zhou, P., Xie, W., Yao, Z., Zhu, Y., Ye, L., & Yu, H. Development of a temperature-responsive yeast cell factory using engineered Gal4 as a protein switch. *Biotechnol. Bioeng.* **115**, 1321-1330 (2018).

4. Xie, W., Lv, X., Ye, L., Zhou, P., & Yu, H. Construction of lycopene-overproducing *Saccharomyces cerevisiae* by combining directed evolution and metabolic engineering. *Metab. Eng.* **30**, 69-78 (2015).
