# Supplementary material for: Fermentative production of Vitamin E tocotrienols in Saccharomyces cerevisiae under cold-shock-triggered temperature control
Source: Nat Commun. 2020 Oct 14;11:5155. doi: 10.1038/s41467-020-18958-9 (PMC7560618; doi:10.1038/s41467-020-18958-9)
Supplement: Supplementary file 2 — Peer Review [file 41467_2020_18958_MOESM2_ESM.pdf]

Reviewer #1 (Remarks to the Author):

The manuscript describes metabolic engineering and gene expression regulation strategies that were successful in obtaining exceptionally high titers of tocotrienols in Saccharomyces cerevisiae. The metabolic engineering strategy was fairly straight-forward and based in part on natural pathways in plants and other photosynthetic organisms. The success of this strategy was due largely to brute force, i.e., testing genes from many sources with and without plastid transit peptides. The gene regulation strategy appears to be rather novel in its design and was effective at obtaining high amounts of biomass prior to "turning on" the engineered tocotrienol pathway in the yeast cells. This strategy enabled high titers of tocotrienols despite the apparent toxicity that these lipophilic molecules confer to growing cells. Overall, the manuscript describes a very commendable effort at producing a novel form of vitamin E tocochromanols in yeast, which has not been previously successful.

Major points:
The authors highly overstate the economic significance of their work. First, tocotrienols may have some novel and desirable nutraceutical and chemical properties, but it is unclear how significant these potential markets are. Tocotrienols can be obtained from palm oil distillates at a relatively low cost. Still, they are not widely used commercially. The commercial antioxidant market is dominated by alpha-toccopherol because, in contrast to tocotrienols, they are readily absorbed by human cells and can be isolated and chemically methylated at a low cost from source such as soybean oil distallates. Second, the authors push the idea that because current sources of tocotrienols are plants, this is somehow not economical compared to a yeast source. This is a fallacy. Oil palm is one of the most productive oil crops in the world. Palm oil is plentiful and cheap. The vitamin E/tocotrienol fraction can be easily obtained by distillation from the oil. It is a co-product that is part of a larger value chain from palm fruits. The authors also do not cite metabolic engineering efforts that have led to elevated levels of tocotrienols in soybean, which does not normally produce this vitamin E form. These publications include DOI: 10.1016/j.ymben.2005.05.005 and DOI: 10.1016/j.ymben.2019.10.005

Other points:
The manuscript is a bit difficult to read and comprehend because so many different gene combinations were tested. As such, it takes a considerable amount of effort for the reader to remember what all of the different constructs are.

Supplementary Table 1 mentions soybean "HGGT". Soybean seeds do not normally make tocotrienols, and I know of no demonstration that soybean has a functional HGGT. I think that this gene is really a variant HPT/VTE2.

Figures 2 through 7 do not provide any information in their legends about numbers of replications, nature of replicates, and what statistical analysis methods were used to obtain the error shown in bar graphs.

The legend for Figure 1 has no information about what the numbers correspond to for the labels of chromatogram peaks in Figure 1b.

As far as I can tell, the authors do not acknowledge that the gamma-tocopherol methyltransferase gene does not work particularly well in yeast. In plant metabolic engineering efforts, overexpression of the two methyltransfeases are very effective in converting nearly all of the tocopherol to the more desired alpha form. This is not the case for yeast in the results presented here.

The analytical methods are not well-described. The authors indicate that UV absorption was used for the HPLC detection. Are they sure about this? Normally fluorescence detection is used. Where did they get their tocotrienol standards? What are the response factors for the tocotrienols? I assume that each form has a different UV response.

The authors should be a bit careful to not use "vitamin E" too loosely to describe tocotrienols. Many nutritionists believe alpha-tocopherol is the only true vitamin E because it is the most readily absorbed form by human cells. As such, the term "vitamin E tocochromanols" is often used to describe tocotrienols.

Line 382 "...MGGBQ and DMGGBQ were almost depleted..." Figure 6B is referred to, but I see no figure with data that shows this. Maybe an HPLC chromatogram showing this would be effective for conveying this versus other prototypes that had high levels of MGGBQ and DMGGBQ.

Reviewer #2 (Remarks to the Author):

Comments on the manuscript of Shen et al. Nature Commun Vitamin E tocotrienols in S cerevisiae

The authors describe the metabolic engineering of yeast (Saccharomyces cerevisiae) to produce the vitamin E compound tocotrienol.
Background: The term vitamin E encompasses a group of 8 different lipid-soluble compounds, 4 tocopherols and 4 tocotrienols, which can be distinguished by different levels of saturation in the prenyl side chain and in the grade of methylation of the tocochromanol ring. Vitamin E is an antioxidant and humans have to take it up with their nutrition. Vitamin E compounds are normally found only in photosynthetic organisms (prokaryotic cyanobacteria, many eukaryotic plants especially grasses/cereals and palms). The various tocopherols and tocotrienols can be extracted from these plant oils. An especially rich source of tocotrienols are palm oil or grass kernels (wheat). (racemic) Tocopherols are produced in large amounts by chemical synthesis (> 10 000 tons annually) while tocotrienols are not.
Importance: The debate on putative positive or negative influences of vitamin E supplementation in human or animal health is still on. As well is the debate on ecological concerns regarding oil palm plantations. Therefore, fermentative production of single vitamin E compounds would be welcome and could open a field for an ecologically agreeable production (albeit with genetically modified microorganisms). Different health claims are attributed to the single vitamin E compounds. Therefore it is of great interest to have single compound producers instead of extracting and purifiying them from plant oil.
Achievements: The authors present the first case for a eukaryotic non-photosynthetic microorganism (yeast) to produce tocotrienols (a case with a non-photosynthetic prokaryotic E. coli δ-tocotrienol producers was reported in 2008). The authors cloned the necessary genes from various sources and thereby combined the biosynthesis pathways for aromatics and isoprenoids. The fermentative production with recombinant yeast strains reached 7.6 mg/g dry cell weight which is already several orders of magnitude higher than the reported values for E. coli. Production in yeast was improved by applying a novel cold-shock-triggered temperature control to allow, in a two-stage fermentation, production of 320 mg/l of tocotrienols. This is a remarkable achievement.
The content of the manuscript is of general interest for researchers in the field of synthetic biology as it both provides a very good example for successful production of a compound of interest in yeast as well as the cold-shock control system is a clever improvement.
The manuscript in general is well written although the discussion part is a bit on the lengthy side (see below) and some technical details on the cold-shock control should be moved to the Supp Info. The methods are presented in sufficient details to be reproduced. Additional data can be found in the Supp Info part.
Major criticism:
1. The part on the design and construction of the cold-shock control system is a bit lengthy. Especially the part were lycopene production was assayed (lines 442-493, including Fig. 7) does not need to appear in the manuscript here. It would fit into the Supplementary information, and only the most important results should appear in the text of the manuscript.
2. The discussion from line 602 through line 622 is largely redundant to the results section and could be shortened drastically. As well, the description in the discussion from line 657 to 680 could be

shortened without much loss.
Minor criticisms:
Introduction line 85 onwards: the discussion of positive health effects seems a bit biased. Palm oil industry certainly has some commercial interests here. Therefore, please also discuss how conclusive reports on health claims are (are any of these reports financed by palm oil industry?) and quote reports on lack of effect or even negative effects of vitamin E (smoker !) and especially tocotrienols .
Introduction, line 92 onwards: to allow the reader a comparison, please provide numbers for tocotrienol concentrations in plant oil (oil palm, wheat kernel).
Introduction, line 99: quotations on synthetic biology: please be fair to other non-chinese authors (quotations 13-16). I doubt that there are no other contributions worldwide on synthetic biology? And there are other model organisms than yeast!
Results: lines 169 and 217, and Figs 1c and 2: here a quotation on MGGBQ structure from recombinant E. coli is missing (Ghanegaonkar et al. 2012 J Biotechnol). Please include and discuss their results with yours as there are differences.
Lines 190 and line 260: the term „ prokaryotic alga" is outdated as Synechocystis are cyanobacteria. Please correct.
Line 241: „4.01 fold higher": does your statistical analysis allow such a significant number? Isn´t „about 4 fold" more adequate ?
Lines 240-251: „82.8%" and „28.4%" again please provide error margins if you give these pseudo-accurate numbers. How often were these measurements done with how many biological replicates ?
Legends to Figs. 3 and 4. Please provide statistical values here: how many biological and technical replicates. Which program did you use for calculating this ?
Lines 302 and 312, again „2.17 folds" , „95.6%" see remarks above.
Legend to Fig. 5: provide number of biological replicates. Give an explanation for the abbreviation „IAA".
Line 429: „tocotrienols titer was 176.70 mg/l". Again, the value appears to be pseudo-accurate, why not „176.7"? Why do you give the titer per l and not mg/g of cdw? Was the tocotrienol found exclusively in the culture supernatant? Or did you collect the cells and extract the tocotrienols from the cells? Please comment.
Missing literature refs. which should be considered:
C K Sen et al. (2006) Tocotrienols: vitamin E beyond tocopherols. Life Sciences 78:2088-98.
S Ghanegaonkar et al. (2012) J Biotechnology 164:238-247.

Reviewer #3 (Remarks to the Author):

In this work, Shen et al. successfully assembled a heterologous biosynthetic pathway for tocotrienol production in S. cerevisiae, based on expression of enzymes from A. thaliana and Synechocystis sp. PCC6803, and demonstrated production of α-, β-, γ-, and δ-tocotrienols. Tocotrienol titers were improved by overexpression of key enzymes, truncation of enzyme sequences to remove putative transit peptides, and enhancement of precursor supply. High density fermentation with the strain initially constructed was limited by slow growth, so a cold-shock-triggered expression system based on a previously characterized temperature-sensitive Gal4 mutant was implemented to control expression of the pathway enzymes. The resulting strain showed enhanced biomass formation and higher tocotrienol titers in a two-stage fed-batch fermentation.

The authors combine several metabolic engineering strategies to improve titers of tocotrienols and present clear results regarding the impact of each improvement during strain engineering. While the manuscript illustrates significant work in strain optimization for tocotrienol production and a useful new application of the temperature-sensitive Gal4 system, it appears to lack the novelty and broad applicability to the field required for Nature Communications and may be more suitable for publication elsewhere.

In the abstract, the authors state that “this is the first report on biosynthesis of tocotrienols by engineered yeast”. However, another group has recently published a report on δ-tocotrienol production in S. cerevisiae (Sun, H. et al., J. Agri. Food Chem., 2020 https://doi.org/10.1021/acs.jafc.0c00294) with reported titers up to 4.1 mg/L. In the manuscript under review, Shen et al. have achieved higher titers of tocotrienols than those reported by Sun et. al, as well as producing α- β- and γ-tocotrienols, along with δ-tocotrienol. They have also carried out more extensive studies in strain optimization to reduce buildup of pathway intermediates. However, the initial strategies used to achieve or improve tocotrienol production are similar between the groups (utilizing HPT from Synechochystis sp. PCC6803, increasing supply of precursors from the MVA pathway) and in low density or shake flask culture, tocotrienol production levels appear to be on the same order of magnitude. Additionally, the pathway for tocotrienol production in A. thaliana has been studied, so the enzymatic conversions required to produce α- β- and γ-tocotrienols were known and were not newly elucidated for this manuscript. The group’s work on expression of the truncated A. thaliana enzymes could provide novel information, but the discussion and work in this area is limited.

The design of the strain which enables high-density growth of S. cerevisiae while still maintaining high tocotrienol production appears to be the most novel aspect of the work. As cited in the manuscript, details on use of the temperature-sensitive Gal4 system have already been published by this group (Zhou, P. et al. Biotechnol. Bioeng. 115, 1321-1330 (2018)), showing its usefulness for two-stage fermentation to enhance lycopene production. In the current manuscript, to allow enhanced expression of pathway enzymes, the authors use the temperature shift to induce expression of Gal4, instead of directly controlling expression of pathway enzymes with Gal4M9. This is a useful modification of the system to allow strong expression of the pathway enzymes, as well as continued expression after returning to 30 C, but does not appear to represent a significant advancement from previous work.

Reviewer #1 (Remarks to the Author):

The manuscript describes metabolic engineering and gene expression regulation strategies that were successful in obtaining exceptionally high titers of tocotrienols in *Saccharomyces cerevisiae*. The metabolic engineering strategy was fairly straight-forward and based in part on natural pathways in plants and other photosynthetic organisms. The success of this strategy was due largely to brute force, i.e., testing genes from many sources with and without plastid transit peptides. The gene regulation strategy appears to be rather novel in its design and was effective at obtaining high amounts of biomass prior to "turning on" the engineered tocotrienol pathway in the yeast cells. This strategy enabled high titers of tocotrienols despite the apparent toxicity that these lipophilic molecules confer to growing cells. Overall, the manuscript describes a very commendable effort at producing a novel form of vitamin E tocochromanols in yeast, which has not been previously successful.

Response: The authors thank the reviewer for the positive comments.

Major points:

The authors highly overstate the economic significance of their work. First, tocotrienols may have some novel and desirable nutraceutical and chemical properties, but it is unclear how significant these potential markets are. Tocotrienols can be obtained from palm oil distillates at a relatively low cost. Still, they are not widely used commercially. The commercial antioxidant market is dominated by alpha-tocopherol because, in contrast to tocotrienols, they are readily absorbed by human cells and can be isolated and chemically methylated at a low cost from source such as soybean oil distillates. Second, the authors push the idea that because current sources of tocotrienols are plants, this is somehow not economical compared to a yeast source. This is a fallacy. Oil palm is one of the most productive oil crops in the world. Palm oil is plentiful and cheap. The vitamin E/tocotrienol fraction can be easily obtained by distillation from the oil. It is a co-product that is part of a larger value chain from palm fruits. The authors also do not cite metabolic engineering efforts that have led to elevated of tocotrienols in soybean, which does not normally produce this vitamin E form. These publications include DOI: 10.1016/j.ymben.2005.05.005 and DOI: 10.1016/j.ymben.2019.10.005

Response: The authors thank the reviewer for the constructive comment. We have carefully revised the Introduction to avoid overstating the economic significance of tocotrienols, and the metabolic engineering efforts in soybean have been cited as suggested. Meanwhile, the sentence "The current market supply of tocotrienols mainly relies on extraction from oil palm, and is therefore largely limited by the source" has been deleted from the Discussion section.

Shown below are the first two paragraphs of Introduction in the revised manuscript: "Vitamin E, an essential nutrient in the human diet, is a generic term that refers to α, β, γ, δ-tocopherols and tocotrienols, all of which are amphipathic molecules with a hydrophobic isoprenoid-derived hydrocarbon tail and a polar aromatic head obtained from the shikimate pathway[1]. The major structural difference of tocotrienols from tocopherols lies in the unsaturated isoprenoid side chain containing three double bonds, which would potentially enhance their deeper penetration into tissues that have saturated fatty layers such as the brain and liver[2]. Similar to α-tocopherol, the most extensively studied vitamin E form, tocotrienols are excellent antioxidants[3, 4]. Moreover, studies have shown some unique functions associated to tocotrienols that are often not exhibited by tocopherols, such as lowering cholesterol by inhibiting the activity of 3-hydroxy-3-methylglutaryl-coenzyme A (HMG-CoA) reductase[5],

neuroprotective function by suppressing early activation of c-Src kinase and 12-lipoxygenase[6] and potent radioprotectant against radiation damage[7]. These properties make tocotrienols valuable supplementations to the current α-tocopherol-based products.

Although racemic α-tocopherol has been chemically synthesized since 1938[8], commercial synthesis of tocotrienols has not been achieved yet, ascribed to the complicated steps, low yields and purity[9]. In nature, vitamin E is exclusively synthesized in oxygenic photosynthetic organisms[10, 11], and tocotrienols are generally found in the seeds of monocotyledons and fruits of some dicotyledons[12]. Currently, natural tocotrienols are mainly derived from crude palm oil which contains up to 800 mg/kg of tocotrienols[13]. But over-planting of oil palm has raised ecological concerns due to its damage to biodiversity and the environment[14]. Recently, metabolic engineering efforts in soybean seeds have turned soybean into a viable platform for producing high levels of vitamin E tocochromanols[15, 16]. However, plant cultivation is generally subject to factors like land occupation and weather or climate changes. In contrast, microbial fermentation does not have these issues and has shorter production cycles, making it an attractive alternative. Recent advances in synthetic biology have enabled heterologous biosynthesis of various natural products in model microorganisms[17, 18, 19, 20]. The synthetic pathway of vitamin E has been determined by mutational analysis and *in vitro* enzyme characterization[21], which lays basis for its heterologous biosynthesis. The first effort on microbial biosynthesis of vitamin E tocochromanols produced 15 μg/g DCW of δ-tocotrienol in engineered *Escherichia coli*[22]. However, the low yield and biosafety concerns with *E. coli* discourage its further application. Considering the GRAS (generally regarded as safe) status of *Saccharomyces cerevisiae* and its outstanding performance in heterologous production of terpenoids and aromatic compounds[23, 24], construction of efficient yeast cell factory may be a viable approach to sustainable tocotrienols production."

Other points:

1. The manuscript is a bit difficult to read and comprehend because so many different gene combinations were tested. As such, it takes a considerable amount of effort for the reader to remember what all of the different constructs are.

Response: Thank you for the helpful suggestion. We have now included a flowchart of yeast strain construction in the Supplementary Material (as Supplementary Fig. 6) to make it easier for the readers to follow.

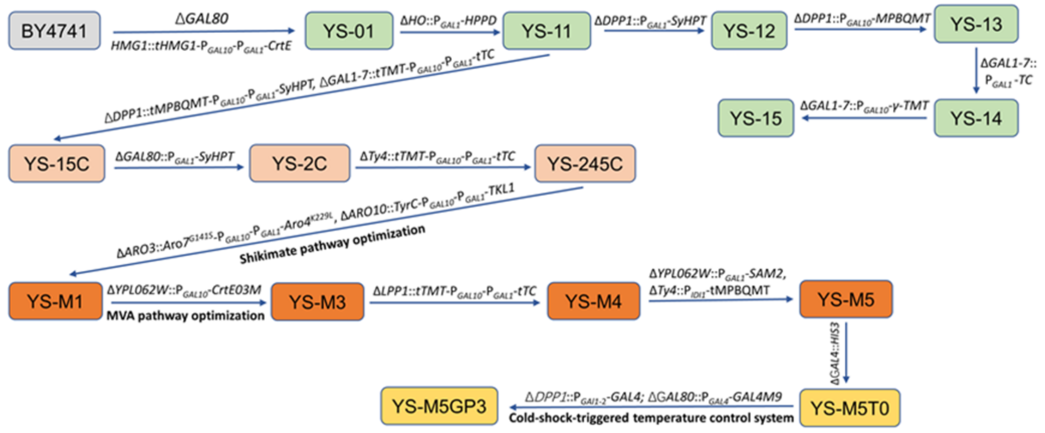

Supplementary Fig. 6 Flowchart of yeast strain construction in this study.

2. Supplementary Table 1 mentions soybean "HGGT". Soybean seeds do not normally make tocotrienols, and I know of no demonstration that soybean has a functional HGGT. I think that this gene is really a variant HPT/VTE2.

Response: Thank you for the correction. We have now revised soybean "HGGT" to "HPT" in Supplementary Table 1.

3. Figures 2 through 7 do not provide any information in their legends about numbers of replications, nature of replicates, and what statistical analysis methods were used to obtain the error shown in bar graphs.

Response: Thank you for the constructive comment. We have now included "All values presented are the means of three biological replicates, and the error bars represent standard deviations." in legends of these figures.

4. The legend for Figure 1 has no information about what the numbers correspond to for the labels of chromatogram peaks in Figure 1b.

Response: Thank you for the helpful suggestion. We have now included the information on the numbers corresponding to the labels of chromatogram peaks in Figure 1b.

5. As far as I can tell, the authors do not acknowledge that the gamma-tocopherol methyltransferase gene does not work particularly well in yeast. In plant metabolic engineering efforts, overexpression of the two methyltransfeases are very effective in converting nearly all of the tocopherol to the more desired alpha form. This is not the case for yeast in the results presented here.

Response: Thank you for the constructive comment. In our study, although the γ-tocopherol methyltransferase worked better after truncation of the transit peptide, it failed to fully convert γ-tocotrienol to α-tocotrienol. We have now revised the manuscript to make it clear that this enzyme did not work as well in the yeast as in the plants. "Although overexpression of the *Arabidopsis* γ-TMT in plant seeds resulted in the near-complete conversion of γ-tocopherol to α-tocopherol[57, 61] and γ-tocotrienol to α-tocotrienol[15], the activity of γ-TMT seemed to be low when expressed in yeast, with γ-tocotrienol remaining as the main product."

6. The analytical methods are not well-described. The authors indicate that UV absorption was used for the HPLC detection. Are they sure about this? Normally fluorescence detection is used. Where did they get their tocotrienol standards? What are the response factors for the tocotrienols? I assume that each form has a different UV response.

Response: Thank you for the constructive comment. We detected the tocotrienols samples by UV/VIS detector at 292 nm according to Albermann C. *et al* (Albermann C. *et al. Chembiochem*, 2008, 9(15): 2524-2533.). In our study, this method worked well for the quantification of tocotrienols samples. The tocotrienol standards were purchased from Solarbio (Beijing, China), as described in the Materials and Methods section of the manuscript. Below are the standard curves of α-, γ-, δ- and β-tocotrienols, the data of which are provided in the Source Data file.

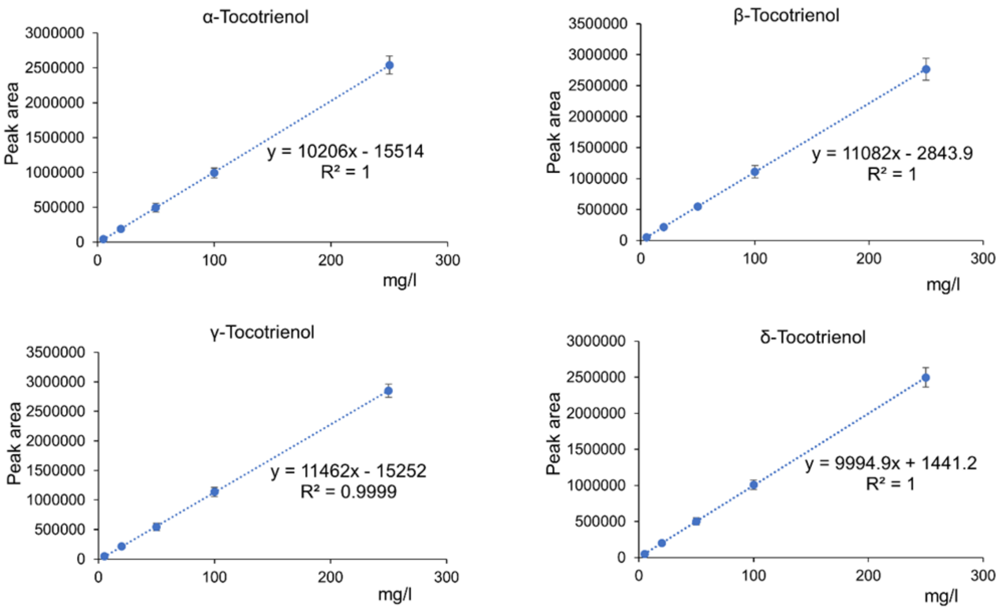

Standard curves of α-, γ-, δ- and β-tocotrienols

7. The authors should be a bit careful to not use "vitamin E" too loosely to describe tocotrienols. Many nutritionists believe alpha-tocopherol is the only true vitamin E because it is the most readily absorbed form by human cells. As such, the term "vitamin E tocochromanols" is often used to describe tocotrienols.

Response: Thank you for the helpful comment. We have now corrected “vitamin E” to “vitamin E tocochromanols” when describing tocotrienols in the revised manuscript.

8. Line 382 "...MGGBQ and DMGGBQ were almost depleted..." Figure 6B is referred to, but I see no figure with data that shows this. Maybe an HPLC chromatogram showing this would be effective for conveying this versus other prototypes that had high levels of MGGBQ and DMGGBQ.

Response: Thank you for the helpful comment. The information on MGGBQ and DMGGBQ contents in YS-M4 has now been included in the Supplementary Material as Supplementary Fig. 3.

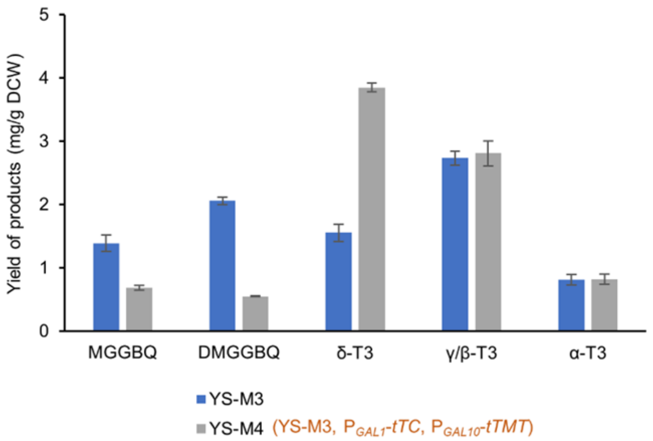

Supplementary Fig. 3 Comparison of metabolites production by YS-M3 and YS-M4. All values presented are the means of three biological replicates, and the error bars represent standard

deviations.

Reviewer #2 (Remarks to the Author):

Comments on the manuscript of Shen *et al*. Nature Commun Vitamin E tocotrienols in *S. cerevisiae*

The authors describe the metabolic engineering of yeast (*Saccharomyces cerevisiae*) to produce the vitamin E compound tocotrienols.

Background: The term vitamin E encompasses a group of 8 different lipid-soluble compounds, 4 tocopherols and 4 tocotrienols, which can be distinguished by different levels of saturation in the prenyl side chain and in the grade of methylation of the tocochromanol ring. Vitamin E is an antioxidant and humans have to take it up with their nutrition. Vitamin E compounds are normally found only in photosynthetic organisms (prokaryotic cyanobacteria, many eukaryotic plants especially grasses/cereals and palms). The various tocopherols and tocotrienols can be extracted from these plant oils. An especially rich source of tocotrienols are palm oil or grass kernels (wheat). (racemic) Tocopherols are produced in large amounts by chemical synthesis (> 10 000 tons annually) while tocotrienols are not.

Importance: The debate on putative positive or negative influences of vitamin E supplementation in human or animal health is still on. As well is the debate on ecological concerns regarding oil palm plantations. Therefore, fermentative production of single vitamin E compounds would be welcome and could open a field for an ecologically agreeable production (albeit with genetically modified microorganisms). Different health claims are attributed to the single vitamin E compounds. Therefore it is of great interest to have single compound producers instead of extracting and purifying them from plant oil.

Achievements: The authors present the first case for a eukaryotic non-photosynthetic microorganism (yeast) to produce tocotrienols (a case with a non-photosynthetic prokaryotic E. coli [illegible]-tocotrienol producers was reported in 2008). The authors cloned the necessary genes from various sources and thereby combined the biosynthesis pathways for aromatics and isoprenoids. The fermentative production with recombinant yeast strains reached 7.6 mg/g dry cell weight which is already several orders of magnitude higher than the reported values for E. coli. Production in yeast was improved by applying a novel cold-shock-triggered temperature control to allow, in a two-stage fermentation, production of 320 mg/l of tocotrienols. This is a remarkable achievement.

The content of the manuscript is of general interest for researchers in the field of synthetic biology as it both provides a very good example for successful production of a compound of interest in yeast as well as the cold-shock control system is a clever improvement.

The manuscript in general is well written although the discussion part is a bit on the lengthy side (see below) and some technical details on the cold-shock control should be moved to the Supp Info. The methods are presented in sufficient details to be reproduced. Additional data can be found in the Supp Info part.

Response: The authors thank the reviewer for the positive comments. We have revised the discussion and moved the details on the cold-shock control to Supplementary Material as suggested.

Major criticism:

1. The part on the design and construction of the cold-shock control system is a bit lengthy. Especially the part of lycopene production was assayed (lines 442-493, including Fig. 7) does not

need to appear in the manuscript here. It would fit into the Supplementary information, and only the most important results should appear in the text of the manuscript.

Response: Thank you for the helpful comment. We have now shortened the description on the design and construction of the cold-shock control system as suggested, and moved the text and figures on validation with lycopene production to Supplementary Material. Lines 442-493 have been revised to "To take advantage of temperature control and meanwhile to avoid the adverse effect of limited Gal4M9 activity on tocotrienols production, we redesigned the temperature control system by introducing an additional level of regulation for the P_{GAL}-driven biosynthetic pathway (Fig. 7a). The wild-type Gal4 was placed under control of P_{GAL1} which could only be switched on by the P_{GAL4}-driven Gal4M9. Upon depletion of glucose in the culture medium, P_{GAL4} initiates expression of the temperature-sensitive Gal4M9, which activates the expression of the P_{GAL1}-driven Gal4 when the temperature is changed to 24°C. The accumulated Gal4 can kick start the expression of P_{GAL}-driven genes including its own encoding gene. In this way, a short-time incubation at 24°C would be sufficient to switch on the target synthetic pathway. Considering that the accumulation of Gal4 under a strong *GAL* promoter may become excessive in this signal amplification system, which would impair cell growth[52,53], the suitable promoter strength for proper control of the Gal4 amount was investigated. To weaken P_{GAL1} while maintaining its response to Gal4 binding, truncated P_{GAL1} variants were created by changing the number of Gal4 binding sites in UAS_G, generating P_{GAL1}-2 with 3 sites and P_{GAL1}-3 with 2 sites[54]. Examination of the promoters in the lycopene-producing yeast Ylyc-TS0 [28] finalized P_{GAL1}-3 as the one with appropriate strength to control Gal4 expression, and 5 h of 24°C incubation was found sufficient to deliver temperature control (Supplementary Fig. 4)."

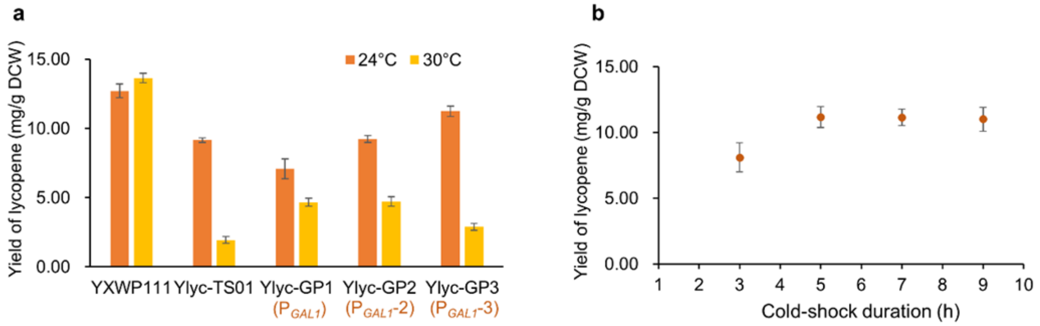

Supplementary Fig. 4 Examination of the cold-shock-triggered temperature control system in the lycopene-producing yeast. **(a)** Optimization of the cold-shock-triggered temperature control system based on the lycopene-producing yeast Ylyc-TS0. Modified P_{GAL1} with appropriate strength was screened for expression control of the wild-type Gal4. **(b)** Determination of the suitable duration of cold shock in Ylyc-GP3. All values presented are the means of three biological replicates, and error bars represent standard deviations.

2. The discussion from line 602 through line 622 is largely redundant to the results section and could be shortened drastically. As well, the description in the discussion from line 657 to 680 could be shortened without much loss.

Response: Thank you for the constructive comment. We have revised and shortened the mentioned discussion paragraphs as suggested.

"Considering that the potential of the heterologous pathway may be limited by insufficient supply of endogenous precursors, the shikimate pathway and MVA pathway were both

strengthened. As expected, comprehensive engineering of the shikimate pathway towards self-sufficient supply of 4-HPP led to higher HGA supply and tocotrienols production as compared to exogenous tyrosine addition, and enhancing GGPP supply further improved tocotrienols synthesis. To match with the reinforced precursor supply and restore metabolic balance, the heterologous pathway was further engineered to convert all accumulated intermediates to tocotrienols, with δ-tocotrienol accumulated as a major component at this stage."

"A cold-shock-triggered temperature control system was then developed by using the temperature-sensitive Gal4M9 to control the expression of wild-type Gal4 so as to amplify the temperature-responsive regulation signal. Different from the traditional regulation systems where the pathway genes are directly regulated by the transcriptional factors, we placed the transcriptional activator Gal4 under control of its temperature-sensitive variant. In this way, the activity reduction of Gal4M9 could be compensated while maintaining its temperature sensitivity, leading to obvious improvement in the product yield. Moreover, a few hours' incubation at the permissive temperature is sufficient in this system, making it more energy-saving than the original system where a low temperature should be maintained for the whole product accumulation stage. Although leaky expression of Gal4 as implied by the accumulation of tocotrienols at the restrictive temperature was observed in shake-flask culture, tocotrienols synthesis was stringently controlled as a two-stage process in fed-batch fermentation possibly due to the more precise control of fermentation parameters. The obviously improved biomass as compared to the glucose-responsive regulation system and much higher tocotrienols production than the original temperature-responsive regulation system demonstrated the stringency and efficiency of this upgraded temperature control system in two-stage fermentation. For further optimization of this system, artificial Gal4 binding sites may be created by directed evolution and used to fine-tune the expression level of the wild-type Gal4, so as to deliver custom-made regulation systems for two-stage fermentation of different pathways."

Minor criticisms:

Introduction line 85 onwards: the discussion of positive health effects seems a bit biased. Palm oil industry certainly has some commercial interests here. Therefore, please also discuss how conclusive reports on health claims are (are any of these reports financed by palm oil industry?) and quote reports on lack of effect or even negative effects of vitamin E (smoker!) and especially tocotrienols.

Response: Thank you for the constructive comment. About discussion on the possible health effects of tocotrienols, we have revised the statement to "Similar to α-tocopherol, the most extensively studied vitamin E form, tocotrienols are excellent antioxidants[3, 4]. Moreover, studies have shown some unique functions associated to tocotrienols that are often not exhibited by tocopherols, such as lowering cholesterol by inhibiting the activity of 3-hydroxy-3-methylglutaryl-coenzyme A (HMG-CoA) reductase[5], neuroprotective function by suppressing early activation of c-Src kinase and 12-lipoxygenase[6] and potent radioprotectant against radiation damage[7].", and only the articles that are not funded by oil palm companies were cited. Vitamin E deficiency was observed in the alveolar fluid of smokers and vitamin E supplementation seemed to be less effective in smokers partly due to the increased oxidative metabolism of vitamin E to the quinone form (Pacht E. R. *et al*. *J. Clin. Invest.,* 1986, 77(3): 789-796.). Excessive supplementation of Vitamin E (α-tocopherol) has been reported to cause

certain adverse reactions, such as increasing risk of hemorrhage and stroke (Reiter *et al*. *Ugeskrift for Laeger*, 2005, 167(49): 4664-4665; Khanna *et al*. *The FASEB Journal*, 2015, 29(3): 828-836). But we could not find reports about the negative effects of tocotrienols, possibly due to the relatively low proportion of studies on tocotrienols. Therefore, we did not describe the negative effects of tocotrienols in the manuscript.

Introduction, line 92 onwards: to allow the reader a comparison, please provide numbers for tocotrienol concentrations in plant oil (oil palm, wheat kernel).

Response: Thank you for the helpful comment. We have provided the tocotrienols concentration in crude palm oil (about 800 mg/kg) in the Introduction.

Introduction, line 99: quotations on synthetic biology: please be fair to other non-Chinese authors (quotations 13-16). I doubt that there are no other contributions worldwide on synthetic biology? And there are other model organisms than yeast!

Response: Thank you for the helpful comment. We have now cited studies on different model organisms contributed by authors from different countries in the revised manuscript, including:

1. Westfall, P. J., Pitera, D. J., Lenihan, J. R., Eng, D., Woolard, F. X., Regentin, R., ... & Fickes, S. Production of amorphadiene in yeast, and its conversion to dihydroartemisinic acid, precursor to the antimalarial agent artemisinin. *Proc. Natl. Acad. Sci.* 109, 655-656 (2012);
2. Jones, J. A., Vernacchio, V. R., Collins, S. M., Shirke, A. N., Xiu, Y., Englaender, J. A., ... & Koffas, M. A. Complete biosynthesis of anthocyanins using *E. coli* polycultures. *mBio*. 8, 3 (2017);
3. Xue, Z., Sharpe, P. L., Hong, S. P., Yadav, N. S., Xie, D., Short, D. R., ... & Pollak, D. W. Production of omega-3 eicosapentaenoic acid by metabolic engineering of *Yarrowia lipolytica*. *Nat. Biotechnol.* 31, 734-740 (2013);
4. Lindberg, P., Park, S., & Melis, A. Engineering a platform for photosynthetic isoprene production in cyanobacteria, using *Synechocystis* as the model organism. *Metab. Eng.* 12, 70-79 (2010).

Results: lines 169 and 217, and Figs 1c and 2: here a quotation on MGGBQ structure from recombinant *E. coli* is missing (Ghanegaonkar et al. 2012. J Biotechnol). Please include and discuss their results with yours as there are differences.

Response: Thank you for the helpful comment. We have quoted and discussed this article in the revised manuscript.

Lines 190 and line 260: the term “prokaryotic alga”is outdated as *Synechocystis* are cyanobacteria. Please correct.

Response: Thank you for the helpful comment. We have corrected “prokaryotic alga” to “cyanobacteria” in revised manuscript.

Line 241: “4.01 fold higher”: does your statistical analysis allow such a significant number? Isn´t “about 4 folds” more adequate?

Lines 240-251: “82.8%” and “28.4%” again please provide error margins if you give these pseudo-accurate numbers. How often were these measurements done with how many biological replicates?

Legends to Figs. 3 and 4. Please provide statistical values here: how many biological and technical replicates. Which program did you use for calculating this?

Lines 302 and 312, again “2.17 folds”, “95.6%” see remarks above.

Legend to Fig. 5: provide number of biological replicates. Give an explanation for the abbreviation “IAA”.

Response: Thank you for the constructive comments. We have corrected the number of significant figures (e.g. 4.01 folds to approximately 4 folds, 82.8% to 83%) and included the statement "All values presented are the means of three biological replicates, and error bars represent standard deviations" in the figure legends. The abbreviations IAA, PAA and 4-HPAA have been explained in the legend of Fig. 5.

Line 429: "tocotrienols titer was 176.70 mg/l". Again, the value appears to be pseudo-accurate, why not "176.7"? Why do you give the titer per l and not mg/g of cdw? Was the tocotrienol found exclusively in the culture supernatant? Or did you collect the cells and extract the tocotrienols from the cells? Please comment.

Response: Thank you for the helpful comment. We have corrected 176.70 mg/l to 176.7 mg/l. We used the titer per l here instead of mg/g of cdw because we wanted to emphasize the influence of cell density on the final titer. The tocotrienols were extracted from the collected cells rather than the culture supernatant. The procedures of tocotrienols extraction are described as "6. Extraction and analysis of products" in Materials and Methods of the manuscript.

Missing literature refs. which should be considered:

C K Sen et al. (2006) Tocotrienols: vitamin E beyond tocopherols. Life Sciences 78:2088-98.

S Ghanegaonkar et al. (2012) J Biotechnology 164:238-247.

Response: Thank you for the helpful comment. We have quoted these references in the revised manuscript.

Reviewer #3 (Remarks to the Author):

In this work, Shen *et al*. successfully assembled a heterologous biosynthetic pathway for tocotrienol production in *S. cerevisiae*, based on expression of enzymes from *A. thaliana* and *Synechocystis* sp. PCC6803, and demonstrated production of α-, β-, γ-, and δ-tocotrienols. Tocotrienol titers were improved by overexpression of key enzymes, truncation of enzyme sequences to remove putative transit peptides, and enhancement of precursor supply. High density fermentation with the strain initially constructed was limited by slow growth, so a cold-shock-triggered expression system based on a previously characterized temperature-sensitive Gal4 mutant was implemented to control expression of the pathway enzymes. The resulting strain showed enhanced biomass formation and higher tocotrienol titers in a two-stage fed-batch fermentation.

The authors combine several metabolic engineering strategies to improve titers of tocotrienols and present clear results regarding the impact of each improvement during strain engineering. While the manuscript illustrates significant work in strain optimization for tocotrienol production and a useful new application of the temperature-sensitive Gal4 system, it appears to lack the novelty and broad applicability to the field required for *Nature Communications* and may be more suitable for publication elsewhere.

Response: The authors thank the reviewer for the positive comments, and have carefully addressed the issues the reviewer raised in a point-to-point manner.

In the abstract, the authors state that "this is the first report on biosynthesis of tocotrienols by engineered yeast". However, another group has recently published a report on δ-tocotrienol production in *S. cerevisiae* (Sun, H. et al., J. Agri. Food Chem., 2020 https://doi.org/10.1021/acs.jafc.0c00294) with reported titers up to 4.1 mg/L. In the manuscript under review, Shen *et al*. have achieved higher titers of tocotrienols than those

reported by Sun *et al*, as well as producing α- β- and γ-tocotrienols, along with δ-tocotrienol. They have also carried out more extensive studies in strain optimization to reduce buildup of pathway intermediates. However, the initial strategies used to achieve or improve tocotrienol production are similar between the groups (utilizing HPT from *Synechochystis* sp. PCC6803, increasing supply of precursors from the MVA pathway) and in low density or shake flask culture, tocotrienol production levels appear to be on the same order of magnitude. Additionally, the pathway for tocotrienol production in *A. thaliana* has been studied, so the enzymatic conversions required to produce α- β- and γ-tocotrienols were known and were not newly elucidated for this manuscript. The group's work on expression of the truncated *A. thaliana* enzymes could provide novel information, but the discussion and work in this area is limited.

Response: The authors thank the reviewer for providing the information on the latest work on δ-tocotrienol biosynthesis published on June 25, 2020. As a matter of fact, we submitted our manuscript in April 2020, and there was no report on tocotrienols production by yeast at that time, we therefore stated that "this is the first report on biosynthesis of tocotrienols by engineered yeast". We have now cited the above-mentioned article in the revised manuscript, and changed this sentence in Abstract to "The success in high-density fermentation of tocotrienols by engineered yeast sheds light on the potential of fermentative production of vitamin E tocochromanols."

By combinatorial engineering of the heterologous synthetic pathway and the endogenous precursor pathways together with development of a cold-shock-triggered temperature-control system, we succeeded to produce tocotrienols at titers of up to 7.6 mg/g DCW (corresponding to 40 mg/L, about 10 times that of the recently reported δ-tocotrienol-producing yeast) in shake-flask cultures and 320 mg/L in high-density fermentation, including the methylated α- and γ-tocotrienols whose microbial synthesis were never reported before. Accordingly, the sentence "This is for the first time biosynthesis of vitamin E is made possible in the nonphotosynthetic yeast." in Discussion has been revised to "This is for the first time biosynthesis of γ- and α-tocotrienols is made possible in nonphotosynthetic organisms."

γ- and α-tocotrienols are reported to have higher oral bioavailability (Yap S. P. *et al. J. Pharm. Pharmacol.*, 2003, 55(1): 53-58), stronger antioxidant activity (Yoshida Y. *et al. J. Biosci. Bioeng.*, 2007, 104(6): 439-445) than δ-tocotrienol. Although the enzymes responsible for producing α- and γ-tocotrienols in plants are known, they had never been heterologously expressed in nonphotosynthetic hosts. As a matter of fact, when we expressed them in yeast, we found they could not work well unless the N-terminal chloroplast transit peptide was properly removed. As suggested by the reviewer, to explore the influence of the N-terminal sequence on the expression of the enzymes, we compared the expression levels of the full-length MPBQMT, TC and γ-TMT and their variants created by truncating different lengths of transit peptide as predicted via fusing them to EGFP and measuring the fluorescence intensity. As shown in the revised Fig. 3c below, obvious enhancement of the fluorescence intensity was observed after removal of the transit peptides, and the signal increased with the increase of the truncated sequence length before the peak value was reached. The higher expression level of the truncated enzymes suggested that the heterologous expression of MPBQMT, TC and γ-TMT in yeast was indeed negatively influenced by the presence of chloroplast transit peptide. For MPBQMT, its expression level reported by the fluorescence intensity was enhanced only by 1-fold, whereas its catalytic activity as judged by DMGGBQ accumulation increased by as much as 9 times. This result indicated that excision of the

transit peptide also improved its catalytic activity. For the other two enzymes, excessive truncation of the N-terminal sequence (longer than 47 aa for TC and 40 aa for γ-TMT) led to reduced activity or even activity loss although the expression level was obviously higher than the intact proteins. These results implied the N-terminal amino acids following the transit peptide may play important roles in the catalytic process. Since the crystal structures of MPBQMT, TC and γ-TMT are not resolved and homologous modeling using SWISS-MODEL failed due to lack of templates with sufficient identity (all below 25%), we were not able to further analyze the influence of the N-terminal sequence on their catalytic activity from the structural view.

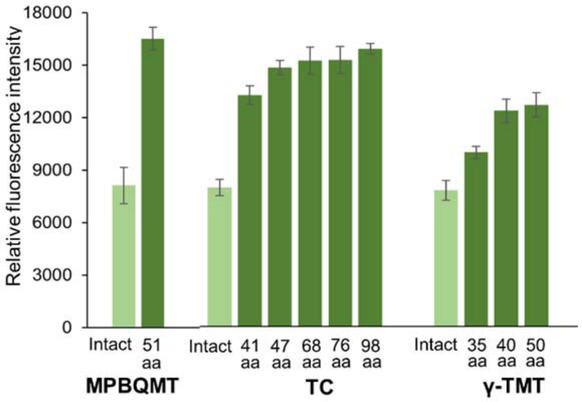

Fig. 3c Changes in relative fluorescence intensity divided by OD_{600} of strains expressing EGFP fused with MPBQMT, TC and γ-TMT before and after truncation of different length of the N-terminal sequence. All values presented are the means of three biological replicates, and error bars represent standard deviations.

The paragraphs related to the expression of truncated proteins in the Results and Discussion have been revised to:

"For MPBQMT, only one possible chloroplast transit peptide with length of 51 aa was predicted, when this N-terminal sequence was excised, the accumulation of DMGGBQ was increased by about 9 times. For TC and γ-TMT, five and three transit peptides with different lengths were predicted respectively. Excision of the 47 aa and 40 aa N-terminal sequences of TC and γ-TMT led to the best results, with 56 % and 67% improvement in the accumulation of corresponding metabolites, respectively (Fig. 3a). To check whether the activity improvement of these enzymes was resulted from enhanced protein expression or altered cellular localization, the influence of transit peptide excision was analyzed using EGFP as a reporter. No change in localization was observed under laser confocal microscopy (Fig. 3b), whereas the fluorescence intensity was obviously increased after removal of the transit peptides. The relative fluorescence intensity increased with the increase of the truncated sequence length before reaching a peak value, and there was about 103%, 86% and 58% increase in the expression levels of the truncated MPBQMT, TC and γ-TMT with highest activities, respectively (Fig. 3c)."

"EGFP-reported expression of the full-length proteins and their variants created by truncating different lengths of transit peptide showed obvious enhancement of the fluorescence intensity

after removal of the transit peptides, and the signal was positively correlated with the truncation length of the N-terminal sequence as well as the amount of DMGGBQ accumulation before the peak values were reached. The higher expression level of the truncated enzymes suggested that the heterologous expression of MPBQMT, TC and γ-TMT in yeast was indeed negatively influenced by the presence of chloroplast transit peptide. For MPBQMT, its expression level reported by the fluorescence intensity was enhanced by only 1-fold, whereas its catalytic activity as judged by DMGGBQ accumulation increased by as much as 9 times. This result indicated that excision of the transit peptide also improved its catalytic activity. For the other two enzymes, excessive truncation of the N-terminal sequence (longer than 47 aa for TC and 40 aa for γ-TMT) led to reduced activity or even activity loss although the expression level was obviously higher than the intact proteins, implying an important role of the N-terminal residues in the catalysis. Surprisingly, the truncated TC and γ-TMT were inactivated when the top-scored transit peptides predicted through ChloroP 1.1 (98 aa and 50 aa, respectively) were removed, whereas excision of 47 aa and 40 aa transit peptides with relatively lower score led to enhanced protein expression. These results suggested that the prediction results should be interpreted with caution, and more than one excision site with high CS score should be included in experimental verification."

The design of the strain which enables high-density growth of *S. cerevisiae* while still maintaining high tocotrienol production appears to be the most novel aspect of the work. As cited in the manuscript, details on use of the temperature-sensitive Gal4 system have already been published by this group (Zhou, P. et al. *Biotechnol. Bioeng*. 115, 1321-1330 (2018)), showing its usefulness for two-stage fermentation to enhance lycopene production. In the current manuscript, to allow enhanced expression of pathway enzymes, the authors use the temperature shift to induce expression of Gal4, instead of directly controlling expression of pathway enzymes with Gal4M9. This is a useful modification of the system to allow strong expression of the pathway enzymes, as well as continued expression after returning to 30 °C, but does not appear to represent a significant advancement from previous work.

Response: The authors thank the reviewer for the positive comment on the temperature control system and we apologize for not clearly describing the cold-shock-triggered temperature control system as a significant advancement from the previous work. Using an environment-responsive transcriptional activator to control the expression of Gal4 instead of the pathway genes is a novel regulation strategy, since generally the transcriptional factors are employed to directly regulate the expression of pathway genes, which is also the case for our previous work. In those cases, the production of the target metabolites would be determined by the activity of the transcriptional factor to a large extent. As a major drawback of the temperature-sensitive Gal4 variant, the dramatically reduced activity limits its efficiency in activating gene expression, and thus leads to limited production of the target metabolite. In particular, for multigene pathways like the tocotrienols synthetic pathway in this work, the product yield would be reduced to a very low level (as shown in Fig. 7b). Introduction of an additional level of regulation by placing the wild-type Gal4 under control of its temperature-sensitive variant could obviously improve the product yield via signal amplification. Another concern with the direct regulation of the pathway genes by temperature-sensitive Gal4 is the requirement of massive cooling water to keep the fermentation at 24°C throughout the production stage. In contrast, the system developed in this work could be activated by a short-term incubation at 24°C and kept active after the temperature

was switched back to 30°C, and is much more energy-saving. Development of the cold-shock-triggered temperature control system simultaneously solved the problems of limited activity and high energy consumption with the previous work, and is thus a big advancement in terms of industrial applicability. Moreover, the expression level of the wild-type Gal4 could be flexibly adjusted as required according to the length of the pathway by altering the strength of its promoter, delivering custom-made regulation systems for two-stage fermentation. All these features make this cold-shock-triggered temperature control system an outstanding tool for bioprocess using yeast chassis. We have now revised the discussion to better clarify the novelty of this system.

Reviewer #1 (Remarks to the Author):

The authors have sufficiently addressed most of the comments from my previous review. Still, my largest concern about the economic justification of the work was not well-addressed. The additions to the manuscript about the challenges of growing crops, climate change, and the environmental sustainability of oil palm are quite off-base. I do not think that the authors appreciate that the glucose and other sugars that are provided to S. cerevisiae in fermenters are produced from crops, especially corn (starch). So if we cannot grow corn, we will not have the nutrients to put into a fermenter. (Also, while some might question the environmental impact of oil palm production, it is a very complex issue that has valid arguments on both sides.) I think it is better to acknowledge that there are plant-based sources of tocotrienols such as oil palm and engineered soybeans, and the yeast-based system is complementary to these production platforms. I think that is sufficient. The metabolic engineering work on its own is quite laudible/significant. The economic value of the yeast production platform will ultimately be determined by the market place.

Reviewer #2 (Remarks to the Author):

The authors have answered in a sufficient way to my remarks. They have changed the manuscript accordingly, have added literature references, moved parts of the manuscript to the Suppl. Info as suggested and have corrected errors.
Statistical analyses are valid as far as I can tell. The level of detail is sufficient to reproduce the work.
The authors have added a flow-chart to visualize the strain constructions.
I have no further questions or comments.

Reviewer #3 (Remarks to the Author):

The authors have made several revisions to their manuscript to address items in my review. Below are some remaining questions/concerns related to the revised manuscript:

The wording of the manuscript was updated in several areas to address the fact there is now another published report of tocotrienol production in yeast (removing references to this manuscript as the first report of tocotrienol production in yeast, etc). I would also suggest citing the reference from Sun et al. in the introduction of the paper, around the point when the merits of implementing the pathway in E. coli versus S. cerevisiae are discussed (lines 108-113).

In the response to reviewers, the authors state "Although the enzymes responsible for producing α- and γ-tocotrienols in plants are known, they had never been heterologously expressed in non-photosynthetic hosts". However, there are reports of the successful expression of both γ-tocopherol methyltransferase (DOI: 10.1126/science.282.5396.2098) and tocopherol cyclase (https://doi.org/10.1073/pnas.182330899) from A. thaliana in E. coli, based on enzymatic assay. In both cases, a predicted transit peptide length was stated and would be worth comparing to the results obtained in this work.

Minor changes:
Lines 269-270: Break into two sentences "…transit peptide with a length of 51 aa was predicted. When this N-terminal sequence…"

Line 299, Line 305: Should be "2.2 fold" / "1.5 fold" instead of "2.2 folds" / "1.5 folds"

Line 577: "…negatively influenced by the presence of the chloroplast transit peptide…"

Response to Reviewers' Comments

Reviewer #1 (Remarks to the Author):

The authors have sufficiently addressed most of the comments from my previous review. Still, my largest concern about the economic justification of the work was not well-addressed. The additions to the manuscript about the challenges of growing crops, climate change, and the environmental sustainability of oil palm are quite off-base. I do not think that the authors appreciate that the glucose and other sugars that are provided to S. cerevisiae in fermenters are produced from crops, especially corn (starch). So if we cannot grow corn, we will not have the nutrients to put into a fermenter. (Also, while some might question the environmental impact of oil palm production, it is a very complex issue that has valid arguments on both sides.) I think it is better to acknowledge that there are plant-based sources of tocotrienols such as oil palm and engineered soybeans, and the yeast-based system is complementary to these production platforms. I think that is sufficient. The metabolic engineering work on its own is quite laudible/significant. The economic value of the yeast production platform will ultimately be determined by the market place.

Response: The authors thank the reviewer for the positive comments and helpful suggestion. As suggested, we have deleted the sentences "But over-planting of oil palm has raised ecological concerns due to its damage to biodiversity and the environment[14]" and "However, plant cultivation is generally subject to factors like land occupation and weather or climate changes. In contrast, microbial fermentation does not have these issues and has shorter production cycles, making it an attractive alternative".

Reviewer #2 (Remarks to the Author):

The authors have answered in a sufficient way to my remarks. They have changed the manuscript accordingly, have added literature references, moved parts of the manuscript to the Suppl. Info as suggested and have corrected errors. Statistical analyses are valid as far as I can tell. The level of detail is sufficient to reproduce the work. The authors have added a flow-chart to visualize the strain constructions. I have no further questions or comments.

Response: The authors thank the reviewer for the positive comments.

Reviewer #3 (Remarks to the Author):

The authors have made several revisions to their manuscript to address items in my review. Below are some remaining questions/concerns related to the revised manuscript: The wording of the manuscript was updated in several areas to address the fact there is now another published report of tocotrienol production in yeast (removing references to this manuscript as the first report of tocotrienol production in yeast, etc). I would also suggest citing the reference from Sun et al. in the introduction of the paper, around the point when the merits of implementing the pathway in E. coli versus S. cerevisiae are discussed (lines 108-113).

Response: The authors thank the reviewer for the positive comments and helpful suggestion. As suggested, we have now included the sentence "De novo biosynthesis of δ-tocotrienol in *S. cerevisiae* has first been reported in June 2020[24], with a titer of 4.1 mg/L." in the Introduction after "construction of efficient yeast cell factory may be a viable approach to sustainable tocotrienols production."

In the response to reviewers, the authors state "Although the enzymes responsible for producing α- and γ-tocotrienols in plants are known, they had never been heterologously expressed in non-photosynthetic hosts". However, there are reports of the successful expression of both γ-tocopherol methyltransferase (DOI: 10.1126/science.282.5396.2098) and tocopherol cyclase (https://doi.org/10.1073/pnas.182330899) from A. thaliana in E. coli, based on enzymatic assay. In both cases, a predicted transit peptide length was stated and would be worth comparing to the results obtained in this work.

Response: The authors thank the reviewer for the correction. We are sorry that we ignored that the γ-tocopherol methyltransferase and tocopherol cyclase have both been heterologously expressed in *E. coli* for functional analysis. We have now included the comparison of predicted transit peptide length of γ-TMT and TC reported in these literatures and our work in the Discussion. "In addition, the transit peptides previously predicted for TC and γ-TMT (74 aa and 47 aa, respectively) which were removed before their functional expression in *E. coli* [57, 59] were not listed as high-score by the software we used. All these results suggested that the prediction results should be interpreted with caution, and more than one excision site with high CS score should be included in experimental verification."

Minor changes:

Lines 269-270: Break into two sentences "…transit peptide with a length of 51 aa was predicted. When this N-terminal sequence…"

Response: Revised as suggested.

Line 299, Line 305: Should be "2.2 fold" / "1.5 fold" instead of "2.2 folds" / "1.5 folds"

Response: Revised as suggested.

Line 577: "…negatively influenced by the presence of the chloroplast transit peptide…"

Response: Revised as suggested.
